# Down-regulation of tomato *PHYTOL KINASE* strongly impairs tocopherol biosynthesis and affects prenyllipid metabolism in an organ-specific manner

**DOI:** 10.1093/jxb/erv504

**Published:** 2015-11-23

**Authors:** Juliana Almeida, Mariana da Silva Azevedo, Livia Spicher, Gaétan Glauser, Katharina vom Dorp, Luzia Guyer, Andrea del Valle Carranza, Ramón Asis, Amanda Pereira de Souza, Marcos Buckeridge, Diego Demarco, Cécile Bres, Christophe Rothan, Lázaro Eustáquio Pereira Peres, Stefan Hörtensteiner, Félix Kessler, Peter Dörmann, Fernando Carrari, Magdalena Rossi

**Affiliations:** ^1^Departamento de Botânica, Instituto de Biociências, Universidade de São Paulo, Rua do Matão, 277, 05508-900, São Paulo, Brazil; ^2^Departamento de Ciências Biológicas, Escola Superior de Agricultura ‘Luiz de Queiroz’, Universidade de São Paulo, Av. Pádua Dias, 11, CP 09, 13418-900, Piracicaba, Brazil; ^3^Laboratory of Plant Physiology, University of Neuchâtel, 2000 Neuchâtel, Switzerland; ^4^Neuchâtel Platform of Analytical Chemistry, University of Neuchâtel, 2000 Neuchâtel,Switzerland; ^5^Institute of Molecular Physiology and Biotechnology of Plants, University of Bonn, D-53115 Bonn, Germany; ^6^Institute of Plant Biology, University of Zurich, Zollikerstrasse 107, CH-8008 Zurich, Switzerland; ^7^CIBICI, Facultad de Ciencias Químicas, Universidad Nacional de Córdoba, CC 5000, Córdoba, Argentina; ^8^INRA and Université de Bordeaux, UMR 1332 Biologie du Fruit et Pathologie, F-33140 Villenave d’Ornon, France; ^9^Instituto de Biotecnología, Instituto Nacional de Tecnología Agropecuaria and Consejo Nacional de Investigaciones Científicas y Técnicas, PO Box 25, B1712WAA, Castelar, Argentina

**Keywords:** Carotenoids, chlorophyll, phytol, phytol kinase, prenyllipids, *Solanum lycopersicum*, tocopherol, tomato, vitamin E.

## Abstract

Tocopherol, a compound with vitamin E (VTE) activity, is a conserved constituent of the plastidial antioxidant network in photosynthetic organisms. The synthesis of tocopherol involves the condensation of an aromatic head group with an isoprenoid prenyl side chain. The latter, phytyl diphosphate, can be derived from chlorophyll phytol tail recycling, which depends on phytol kinase (VTE5) activity. How plants co-ordinate isoprenoid precursor distribution for supplying biosynthesis of tocopherol and other prenyllipids in different organs is poorly understood. Here, *Solanum lycopersicum* plants impaired in the expression of two *VTE5*-like genes identified by phylogenetic analyses, named *SlVTE5* and *SlFOLK*, were characterized. Our data show that while SlFOLK does not affect tocopherol content, the production of this metabolite is >80% dependent on SlVTE5 in tomato, in both leaves and fruits. VTE5 deficiency greatly impacted lipid metabolism, including prenylquinones, carotenoids, and fatty acid phytyl esters. However, the prenyllipid profile greatly differed between source and sink organs, revealing organ-specific metabolic adjustments in tomato. Additionally, VTE5-deficient plants displayed starch accumulation and lower CO_2_ assimilation in leaves associated with mild yield penalty. Taken together, our results provide valuable insights into the distinct regulation of isoprenoid metabolism in leaves and fruits and also expose the interaction between lipid and carbon metabolism, which results in carbohydrate export blockage in the VTE5-deficient plants, affecting tomato fruit quality.

## Highlight

Phytol kinase plays a key role in the regulation of isoprenoid metabolism in an organ-specific manner.

## Introduction

Tocopherols are potent lipid-soluble antioxidants synthesized only by photosynthetic organisms and, together with tocotrienols, are collectively referred to as vitamin E (VTE) compounds (Kamal-Eldin and Appelqvist, 1996; DellaPenna and Pogson, 2006). Since plants are the major source of VTE required for human nutrition, understanding of the mechanisms underlying its synthesis and accumulation in crop species is of great interest (Grusak and DellaPenna, 1999; Fitzpatrick *et al.*, 2012). The antioxidant function of tocopherols relies on their ability to scavenge peroxyl radicals, limiting lipid oxidation of polyunsaturated fatty acids (PUFAs) (Serbinova *et al.*, 1991; Traber and Atkinson, 2008), and also singlet oxygen (^1^O_2_) (Di Mascio *et al.*, 1990; Kaiser *et al.*, 1990; Fukuzawa *et al.*, 1997). In plants, light-driven photosynthetic processes are the main contributors to reactive oxygen species (ROS) production in chloroplasts owing to electron transport chains and photosensitizing molecules such as chlorophyll (Chl) (Edreva, 2005; Demmig-Adams *et al.*, 2014). The delicate equilibrium between ROS production and their detoxification in chloroplast, which determines damage, protection, or signaling response, is controlled by a diversified ROS-scavenging system, including non-enzymatic antioxidant mechanisms (Edreva, 2005; Foyer and Noctor, 2005). Tocopherols, as part of the photoprotective machinery, are particularly involved in controlling the level of ^1^O_2_ in photosystem II (PSII), and the extent of lipid peroxidation in thylakoid membranes especially under stress conditions (Triantaphylidès and Havaux, 2009; Rastogi *et al.*, 2014; Miret and Munné-Bosch, 2015). Beyond photoprotective roles, tocopherol is also involved in seed longevity, seedling germination (Sattler *et al.*, 2004; Mène-Saffrané *et al.*, 2010), and photoassimilate export (Maeda *et al.*, 2006, 2008; Asensi-Fabado *et al.*, 2014); although, for the latter, the precise underlying mechanism remains elusive (Maeda *et al.*, 2014).

**Fig. 1.**
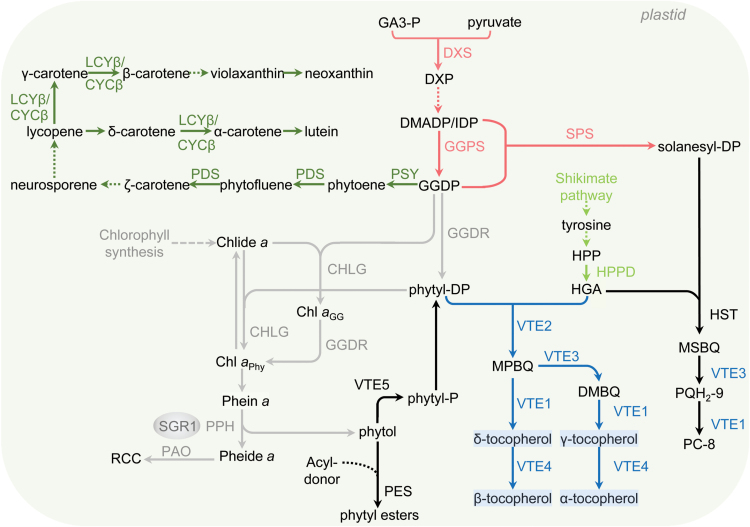
Schematic view of tocopherol biosynthetic and related pathways. The genes are the following: 1-deoxy-d-xylulose-5-P synthase (DXS); geranylgeranyl diphosphate reductase (GGDR); 4-hydroxyphenylpyruvate dioxygenase (HPPD); homogentisate phytyl transferase (VTE2); 2,3-dimethyl-5-phytylquinol methyltransferase (VTE3); tocopherol cyclase (VTE1); γ-tocopherol-C-methyl transferase (VTE4); phytoene synthase (PSY); phytoene desaturase (PDS); chloroplast-speciﬁc β-lycopene cyclase (LCYβ); chromoplast-speciﬁc β-lycopene cyclase (CYCβ); chlorophyll synthase (CHLG); staygreen 1 (SGR1); pheophytinase (PPH); pheophorbide a oxygenase (PAO); phytol kinase (VTE5); farnesol kinase (FOLK); homogentisate solanesyl transferase (HST); solanesyl diphosphate synthase (SPS). Abbreviated intermediate metabolites are: glyceraldehyde 3-phosphate (GA3-P); 1-deoxy-d-xylulose-5-P (DXP); isopentenyl diphosphate (IDP); dimethylallyl diphosphate (DMADP); geranylgeranyl diphosphate (GGDP); hydroxyphenylpyruvate (HPP); homogentisate (HGA); chlorophyllide *a* (Chlide *a*); geranylgeranyl-chlorophyll *a* (Chl *a*_GG_); phytylated chlorophyll *a* (Chl *a*_Phy_); pheophytin *a* (Phein *a*); pheophorbide *a* (Pheide *a*); red chlorophyll catabolite (RCC); 2-methyl-6-geranylgeranylbenzoquinol (MPBQ); 2,3-dimethyl-6-geranylgeranylbenzoquinol (DMBQ); 2-methyl-6-solanyl-1,4-benzoquinol (MSBQ); plastoquinol-9 (PQH2-9); plastochromanol-8 (PC-8).

Accumulation of tocopherol in plant tissues is a tightly controlled process, and several studies determined that tocopherol levels change signiﬁcantly during plant growth and development, as well as in response to environmental stimuli including high light, low temperature, salt, and osmotic stress (Munné-Bosch, 2005; Maeda *et al.*, 2006; Abbasi *et al.*, 2007; Loyola *et al.*, 2012; Quadrana *et al.*, 2013; Eugeni-Piller *et al.*, 2014). Additionally, transgenic approaches have demonstrated that VTE content correlates with the expression of the biosynthesis- and recycling-related genes (reviewed by DellaPenna and Mène-Saffrané, 2011). Tocopherol synthesis occurs in plastids and requires two precursors, a prenyl side chain and a tyrosine catabolite-derived head group (Fig. 1). The prenyl moiety phytyl diphosphate and homogentisate derived from the plastidial 2-C-methyl-d-erythritol 4-phosphate (MEP) and the shikimate pathway, respectively, are condensed by homogentisate phytyl transferase (VTE2), the only enzyme unique for tocopherol synthesis. From this precursor, the four naturally occurring tocopherol forms (α-, β-, γ-, and δ-tocopherol), which vary in the methylation pattern of the chromanol ring, are synthesized via the action of dimethyl-phytylquinol methyl transferase (VTE3), tocopherol cyclase (VTE1), and tocopherol γ-methyl transferase (VTE4). These enzymes are also responsible for the synthesis of the other tocochromanol compounds, which include not only tocotrienols but also plastochromanol (PC-8), a product of plastoquinone (PQ-9) cyclization (Zbierzak *et al.*, 2010).

In addition to the *de novo* synthesis, the phytyl diphosphate precursor may also originate from Chl turnover or degradation, by the release of a phytol moiety from the tetrapyrrole ring. Intriguingly, the precise identity of the tocopherol biosynthesis-related phytol hydrolase remains to be determined. In *Arabidopsis thaliana*, the absence of the known dephytylating enzymes, pheophytinase (PPH) and chlorophyllase (CLH), in triple mutants does not alter seed tocopherol content, whereas seed-specific *PPH*-overexpressing transgenic lines exhibit modestly increased tocopherol levels (Zhang *et al.*, 2014). The hydrolyzed phytol is sequentially phosphorylated by two enzymes, phytol kinase (VTE5) and phytyl-phosphate kinase (VTE6) (Ischebeck *et al.*, 2006; Valentin *et al.*, 2006; vom Dorp *et al.*, 2015). VTE5 has been characterized in Arabidopsis where its mutant allele, *vte5*, causes a substantial reduction of the tocopherol content in seeds and to a lesser extent in leaves (Valentin *et al.*, 2006). Furthermore, based on sequence similarity, a locus encoding a putative VTE5 paralog was identified in Arabidopsis, which further was characterized as a farnesol kinase (FOLK) (Fitzpatrick *et al.*, 2011). However, its involvement in tocopherol biosynthesis was not addressed. So far, VTE5 has only been characterized in Arabidopsis, and its contribution to tocopherol content is largely unknown in other species and organs, such as in edible fleshy fruits. Moreover, the impact of VTE5 deficiency on plant metabolism remains unexplored. *Solanum lycopersicum* is an interesting model species for studying tocopherol metabolism. Besides being an important food crop worldwide, the fruits are a significant source of VTE for the human diet (Chun *et al.*, 2006). Additionally, tomato ripening, which encompasses the conversion of chloroplasts into chromoplasts, couples Chl degradation and an active MEP pathway (Seymour *et al.*, 2013), both sources of the prenyl precursor for tocopherol biosynthesis (Almeida *et al.*, 2015). A previous study on the regulation of tocopherol biosynthesis in this species demonstrated a strong correlation between *VTE5* mRNA levels and the contents of Chl and tocopherol in tomato leaves and fruits, suggesting the contribution of phytol recycling to tocopherol biosynthesis (Quadrana *et al.*, 2013). Moreover, expression analysis of senescence-related tomato mutants suggested that maintenance of the *de novo* phytyl diphosphate synthesis might, at later ripening stages, compensate for the lack of Chl-derived phytol for tocopherol production in fruits (Almeida *et al.*, 2015).

To better understand the extent of the contribution of the VTE5-dependent phytol pathway to tocopherol biosynthesis in source and sink organs, we functionally characterized *VTE5*-like genes in tomato. Tocopherol content was dramatically compromised in both leaves and fruits of *SlVTE5*-knockdown plants. In contrast, analyses of the *folk* mutant genotype ruled out *SlFOLK* as a major contributor to phytol kinase activity required for tocopherol biosynthesis. Additionally, VTE5 deficiency differentially impacts fatty acid phytyl ester and prenyllipid metabolism in fruits and leaves, and also has consequences in photosynthesis and sugar partitioning.

## Materials and methods

### Plant material, growth conditions, and sampling

Seeds of tomato (*Solanum lycopersicum*, cv. Micro-Tom) were obtained from the Laboratory of Hormonal Control of Plant Development (www.esalq.usp.br/tomato). The *folk-1* tomato mutant was isolated from an ethyl methanesulfonate (EMS)-mutagenized Micro-Tom collection from INRA, France. Plants were grown in a greenhouse under automatic irrigation at an average temperature of 25 °C, 11.5h/13h (winter/summer) photoperiod, and 250–350 μmol m^−2^ s^−1^ of incident photoirradiance. Source (the first fully expanded leaf) and sink (the first apical leaf not fully expanded) leaves were sampled. Fruits were harvested at mature green, 1 d after breaker (B+1), 3 d after breaker (B+3), and ripe (B+6) stages at 35, 38, 40, and 43 d after anthesis, respectively. Samples were frozen in liquid N_2_ and stored at –80 °C. All biochemical analyses were performed in the T_1_ generation. For photosynthesis and yield evaluation, an independent experiment in the T_2_ generation was performed. Destructive harvest took place at a point where the largest possible numbers of fruits were ripe without visible over-ripening (15 weeks old) (Vicente *et al.*, 2015). At harvest time, aerial biomass was weighed and all the fruits were counted and weighed.

### Phylogenetic analysis

For phylogenetic analysis, Blastp searches were performed using the protein sequences of *A. thaliana* VTE5 (At5g04490) and FOLK (At5g58560) as queries against the tomato genome (http://solgenomics.net). Homologous sequences from other plant species were retrieved by Blastp from the Phytozome database (http://phytozome.jgi.doe.gov/pz/portal.html). *Nicotiana benthamiana* sequences were obtained from the Sol Genomics Network database (http://solgenomics.net). The sequences were aligned using the MUSCLE package available in the MEGA 5.0 software with default parameters (Tamura *et al.*, 2007), and Neighbor–Joining phylogenies with 5000 bootstrap replications were created with the distances calculated according to the best model indicated by MEGA 5.0.

### Generation of *SlVTE5*-RNAi transgenic lines

Transgenic plants expressing a *SlVTE5*-specific intron-spliced hairpin sequence under the control of the *Cauliflower mosaic virus* 35S promoter were obtained for RNAi-mediated silencing of the Solyc03g071720 locus. A 237bp fragment of *SlVTE5* was amplified by PCR using the primers RNAi-VTE5-F and RNAi-VTE5-R listed in Supplementary Table S1 available at *JXB* online. PCR products were cloned into pENTR/d-TOPO vector (Invitrogen) via directional cloning, and then recombined into the binary vector pK7GWIWG2 (Karimi *et al.*, 2002) to generate pK7GWIWG2(I)-*SlVTE5*. *Agrobacterium*-mediated transformation (strain EHA105) of *S. lycopersicum* was performed according to Pino *et al.* (2010). The presence of the transgene in T_0_, T_1_, and T_2_ kanamycin-resistant plants was detected by PCR in genomic DNA using 35S-right and RNAi-VTE5-R primers (see Supplementary Table S1).

### Identification of the *folk-1* tomato mutant by TILLING

Mutations in *SlFOLK* were identified by screening an EMS-mutagenized tomato population (Just *et al.*, 2013) essentially as described in Okabe *et al.* (2011). TILLING (Targeting Induced Local Lesions In Genomes) unlabeled external primers and internal primers 5′ labeled with IRDye 700 and IRDye 800 dye are listed in Supplementary Table S1 at *JXB* online. Induced point mutations were identified using the mismatch-specific endonuclease ENDO 1. Digested DNA fragments were separated on a Li-Cor DNA analyzer (LI-Cor, Lincoln, NE, USA). The mutation analysis was performed using PARSESNP (Taylor and Greene, 2003) and SIFT (Ng and Henikoff, 2003) software. Homozygous mutant plants were identified by sequencing of the tilled M_3_ family. Phenotypic characterization was performed in M_4_ plants homozygous for the *folk-1* allele using the corresponding segregating individuals homozygous for the *FOLK* wild-type allele as control genotype.

### qPCR analysis

RNA extraction, cDNA synthesis, and real-time quantitative PCR (qPCR) assays were performed as described by Quadrana *et al.* (2013). Primer sequences are listed in Supplementary Table S1 at *JXB* online. qPCRs were performed in a 7500 real-time PCR system (Applied Biosystems) using 2× SYBR Green Master Mix reagent (Applied Biosystems). Expression values were normalized against the geometric mean of two reference genes, *CAC* and *EXPRESSED*, according to Quadrana *et al.* (2013). A permutation test lacking sample distribution assumptions (Pfaffl *et al.*, 2002) was applied to detect statistical differences (*P*<0.05) in expression ratios using the algorithms in the fgStatistics software package (Di Rienzo, 2009).

### Leaf gas exchange and fluorescence measurements

Gas exchange and Chl fluorescence parameters were evaluated in 5-week-old plants using a portable open gas-exchange system incorporating infra-red CO_2_ and water vapor analyzers (LI-6400XT system; Li-Cor) equipped with an integrated modulated Chl fluorometer (LI-6400-40; Li-Cor). Reference [CO_2_] was held at 400 µmol mol^−1^ and the temperature at 25 °C for all measurements. Air humidity inside the leaf chamber was controlled to the externally measured greenhouse relative humidity (50–60%). Carbon assimilation rate (*A*), leaf stomatal conductance (*g*_*s*_), leaf dark respiration (*R*_d_), and fluorescence parameters were measured at a photosynthetic photon flux density (PPFD) of 600 µmol m^−2^ s^−1^ in the first fully expanded leaf between 10:00h and 14:00h. The parameters derived from Chl fluorescence, including light-adapted PSII maximum quantum efficiency (*F′*_v_/*F′*_m_), proportion of open PSII centers (photochemical quenching, *qP*), and PSII operating efficiency (Φ_PSII_), were calculated according to Genty *et al.* (1989).

### Tocopherol, free phytol, and fatty acid phytyl ester quantification

Tocopherols were extracted and measured by HPLC as previously described (Yang *et al.*, 2011). For determination of fatty acid phytyl esters (FAPEs) and free phytol, total lipids were extracted with chloroform according to Lippold *et al.* (2012). Non-polar lipids were purified using chromatography on silica columns (Kieselgel 60; Merck). FAPEs were measured by direct infusion nanospray quadrupole time-of-ﬂight tandem mass spectrometry (Q-TOF-MS/MS; Agilent 6530 Accurate Mass Q-TOF) using methanol:chloroform:300mM ammonium acetate [665:300:35 (v/v/v); Welti *et al.*, 2002] as the solvent system. FAPEs were detected in the positive ion mode by neutral loss scanning for *m*/*z* 278.2974, a fragment characteristic for the phytol moiety. For phytol measurements, the non-polar lipid fraction was silylated and then phytol was quantified by GC-MS as previously described (Lippold *et al.*, 2012).

### Prenylquinone and carotenoid profile

Prenylquinone and related compounds (α-tocopherolquinone, PQ-9, plastoquinol-9, hydroxy-plastoquinone, PC-8, hydroxy-plastochromanol, and ubiquinone-10) were analyzed by a targeted analysis of the lipidomic profile obtained by ultra-HPLC coupled with atmospheric pressure chemical ionization-quadrupole time-of-ﬂight mass spectrometry (UHPLC-APCI-QTOF-MS) as described in Martinis *et al.* (2013) with the following modifications. Briefly, 15mg of lyophilized tissue were exactly weighed and resuspended in 500 μl of tetrahydrofurane:methanol:water 42.5:42.5:15 (v/v/v). The mixture was homogenized using glass beads (1mm in diameter) for 3min at 30 Hz in a tissue lyser. After two rounds of centrifugation (3min, 14 000 *g*, and 4 °C), supernatants were transferred to vials. Prenyllipids were separated on a reverse-phase Acquity BEH C18 column (50×2.1mm, 1.7 μm) under the following conditions: solvent A=water; solvent B=methanol; 80–100% B in 3min, 100% B for 2min, re-equilibration at 90% B for 0.5min. The flow rate was 0.8ml min^–1^ and the injection volume was 2.5 μl. PQ-9 and PC-8 were quantiﬁed based on calibration curves obtained from standard compounds. Data were processed using MassLynx version 4.1 (Waters).

Carotenoids were extracted and detected as described in Almeida *et al.* (2015) using an Agilent 1200 Series HPLC system coupled with a diode array detector on a reverse phase column [Zorbax Eclipse Plus C18 (150 mm×4.6mm, 5 μm), Agilent Technologies]. Compounds were identified at 440nm by their order of elution and absorption spectra (Gupta *et al.*, 2015), and co-migration with authentic standards (all-*trans*-lycopene, all-*trans*-β-carotene, lutein, violaxanthin, neoxanthin, and zeaxanthin). Relative quantification was performed based on chromatografic peak area normalized against sample dry weight.

### Chlorophyll and chlorophyll catabolites

Chl and green catabolites (chlorophyllide, pheophorbide, and pheophytin) were extracted from 10mg of lyophilized tissue during 17h at –20 °C in 90% (v/v) acetone, 10% (v/v) 0.2M TRIS-HCl, pH 8.0, pre-cooled to –20 °C (5ml g^−1^ initial fresh weight). After centrifuging twice (2min, 16 000 *g*, 4 °C), supernatants were analyzed by HPLC as described (Langmeier *et al.*, 1993). Pigments were identified by their absorption spectra at 665nm. For quantification, peak areas were analyzed and referred to calibration curves built from known quantities of standard pigments (Schelbert *et al.*, 2009).

### Quantification of soluble sugars and starch

A 10mg aliquot of the lyophilized samples was extracted five times with 1.5ml of 80% ethanol at 80 °C according to De Souza *et al.* (2013). Combined supernatants were dried under vacuum and re-suspended in 1ml of ultrapure water. To remove pigments, an extraction with 0.5ml of chloroform was performed. Alcohol-soluble sugar quantification was done by high-performance anion exchange chromatography with pulsed amperometric detection (HPAEC/PAD) (Dionex-ICS3000, Dionex). Sugar separation was carried out on a CarboPac PA1 column using isocratic elution of 150mM NaOH with a flow rate of 1ml min^−1^. The calibration curves were prepared using standard solutions of glucose, fructose, and sucrose with a concentration range from 50 μM to 200 μM.

For starch quantification, the dried insoluble material obtained after ethanol extraction was treated with α-amylase (120U ml^−1^, Megazyme) from *Bacillus licheniformis* and amyloglucosidase (30U ml^−1^, Megazyme) from *Aspergillus niger* according to Amaral *et al.* (2008). The glucose content obtained after starch hydrolysis was determined from extract aliquots of 20 μl and 50 μl for leaves and fruits, respectively, after an incubation with glucose oxidase/peroxidase and d-4-aminoantipirine (GOD/POD). Absorbance of quinoneimine dye, which is directly proportional to glucose concentration, was measured spectrophotometrically using an ELISA-type microplate reader at 490nm. A standard curve was prepared using high purity glucose solution (Sigma) ranging from 2.5 µg ml^−1^ to 12.5 µg ml^−1^.

### Trolox equivalent antioxidant capacity (TEAC) assay

The antioxidant capacity of non-polar extracts was assayed as previously described (Re *et al.*, 1999), with minor modiﬁcations. The pre-formed radical 2,2′-azin-obis-(3-ethylbenzothiazoline-6-sulfonic acid) (ABTS·^+^) was produced by oxidation of 7mM ABTS with potassium persulfate (2.45mM ﬁnal concentration) dissolved in ultrapure water. The mixture was incubated in the dark at room temperature for 12–16h before use. The ABTS·^+^ solution was diluted with ethanol and adjusted to 0.70±0.02 absorbance units at 734nm. A 50 µl aliquot of diluted extract or Trolox standard was mixed with 150 µl of diluted ABTS·^+^ solution, and the absorbance was read at 734nm after 10min at 30 ºC. The ABTS·^+^ antioxidant capacity was reported as µmol of TEAC per gram of sample on a dry weight basis by comparison with a Trolox standard curve (0.015–0.50mM). Analyses were run in triplicate at two dilutions for a total of six assays per sample.

### Transmission electron microscopy

Leaf segments were ﬁxed at 4 °C in Karnovsky’s solution [2.5% glutaraldehyde, 2% (v/v) paraformaldehyde in 0.1M sodium phosphate buffer pH 7.2] for 24h. After washing in buffer, the samples were post-ﬁxed in buffered 1% (w/v) osmium tetroxide, washed, dehydrated in a graded series of acetone, and embedded in Spurr resin. The resin was polymerized at 60 ºC. Ultrathin sections were stained with saturated uranyl acetate (Watson, 1958) and lead citrate (Reynolds, 1963), and observed using a Zeiss EM 900 transmission electron microscope.

### Data analyses

Statistical analyses were performed using R statistical software (www.r-project.org). To determine significant differences between the transgenic lines and the control, data were analyzed by *t*-test or ANOVA followed by a Dunnett’s multiple comparison test with the level of significance set to 0.05.

## Results

### Tomato tocopherol contents are highly dependent on *SlVTE5* but not on *SlFOLK*

By using the *A. thaliana* VTE5 protein sequence (At5g04490; Valentin *et al.*, 2006) as query, a survey for homologous sequences in the *S. lycopersicum* genome was performed in the Solanaceae Genomics Network (http://solgenomics.net/). Two loci were identified, Solyc03g071720 and Solyc09g018510. In order to establish the orthology relationships, a phylogenetic analysis was performed with VTE5 homologous protein sequences of 14 flowering species with completely sequenced genomes. The tree revealed two clades whose topology coincided with the established phylogenetic relationships between the analyzed species. One clade contains the Arabidopsis VTE5 protein sequence (Valentin *et al.*, 2006) that clustered together with Solyc03g071720. The other clade groups At5g58560, an earlier proposed VTE5 paralog that was further identified as a farnesol kinase (FOLK; Fitzpatrick *et al.*, 2011), together with Solyc09g018510. This analysis displayed VTE5 and FOLK proteins as sister clades, and the respective genes were named *SlVTE5* and *SlFOLK* (see Supplementary Fig. S1 at *JXB* online). Both genes showed similar expression patterns, with the highest mRNA levels found in green tomato tissues (Supplementary Fig. S2).

**Fig. 2.**
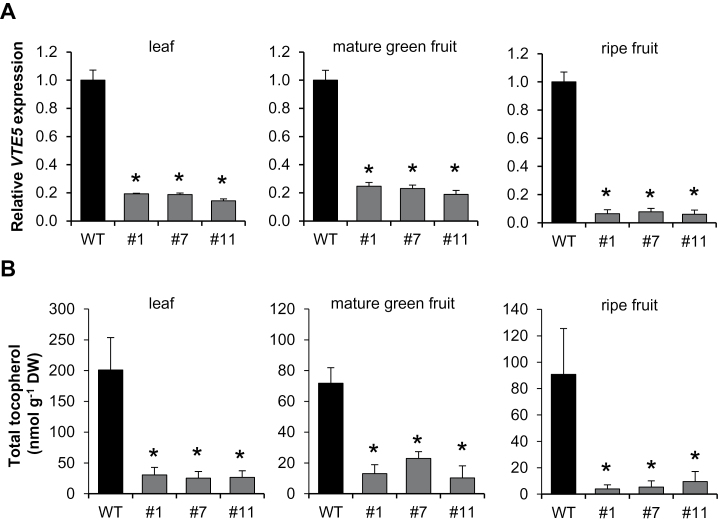
Down-regulation of *SlVTE5* expression and tocopherol content in *SlVTE5*-RNAi transgenic lines. (A) Relative expression of the *SlVTE5* gene in the wild-type (WT) and *SlVTE5*-RNAi lines (#1, #7 and #11). Data are means ±SEM of five biological replicates. The asterisks denote statistically signiﬁcant differences (permutation test, *P*<0.05). (B) Total tocopherol was measured in leaves, mature green, and ripe fruits of *SlVTE5*-RNAi lines. Data represent the mean ±SD of five biological replicates. The asterisks denote significant differences between the WT and the transgenic lines (ANOVA/Dunnett’s test, *P*<0.05).

In order to obtain experimental evidence regarding SlVTE5 function in tocopherol biosynthesis, transgenic *SlVTE5*-knockdown plants were generated by RNAi-mediated silencing. Out of eight primary transformants that showed reduced levels of *SlVTE5* mRNA (see Supplementary Fig. S3 at *JXB* online), three lines with a reduction of >80% were selected for further analyses; *SlVTE5*-RNAi#1, *SlVTE5*-RNAi#7, and *SlVTE5*-RNAi#11 (Fig. 2A). Under normal growth conditions, these transgenic lines exhibited no evident morphological alterations and an apparently unaltered pattern of fruit degreening (Supplementary Fig. S3B).

HPLC analysis revealed that down-regulation of *SlVTE5* resulted in a dramatic reduction (80–90%) of total tocopherol contents both in leaves and in fruits (Fig. 2B). Overall, no differences in tocopherol composition were observed (Supplementary Table S2 at *JXB* online) except for line #7 at the mature green stage. Notably, we detected only traces of tocotrienols in *SlVTE5*-RNAi lines (data not shown). These results ruled out the possibility that depletion of tocopherols could be compensated by tocotrienol production in these plants.

**Fig. 3.**
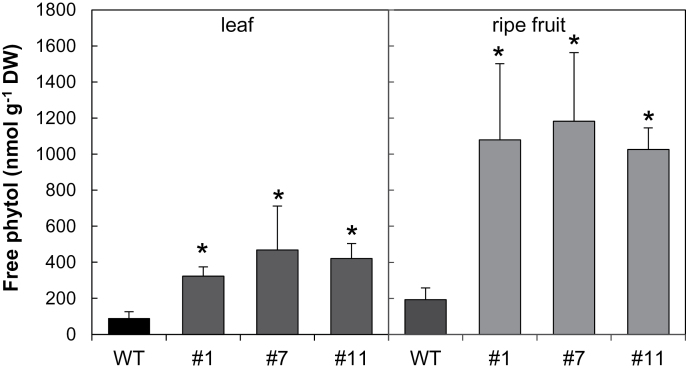
Free phytol content in leaves and ripe fruits of the *SlVTE5*-RNAi transgenic lines. Data represent the mean ±SD of at least three biological replicates. The asterisks denote significant differences between the wild-type (WT) and the transgenic lines (ANOVA/Dunnett’s test, *P*<0.05).

The VTE5 deﬁciency in transgenic lines would be expected to increase free phytol content (Valentin *et al.*, 2006). We therefore assayed the amount of this metabolite by GC-MS. While in mature leaves and ripe fruits of wild-type tomato plants the amount of free phytol ranged from 100 nmol g^−1^ DW to 190 nmol g^−1^ DW, in the counterparts from *SlVTE5*-RNAi lines this prenyl alcohol accumulated four to five times more (Fig. 3). Interestingly, the molar amount of free phytol that accumulated in leaves was of the same order of magnitude as the reduction observed in total tocopherol contents. Strikingly, the increase in the amounts of free phytol in transgenic ripe fruits was 10 times higher than the decrease in tocopherol (Supplementary Table S3 at *JXB* online), suggesting differential regulation in the response of phytol metabolism perturbations in source and sink tomato organs.

**Fig. 4.**
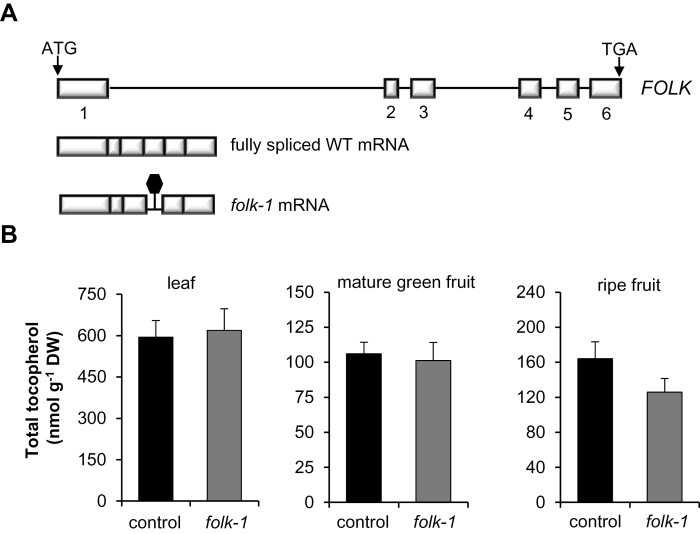
Total tocopherol content in the *folk-1* mutant. (A) Diagram showing the *SlFOLK* gene and fully spliced mRNA found in the wild-type (WT) and abnormally spliced mRNA found in the *folk-1* mutant. Boxes and solid lines represent exons and introns, respectively. The premature stop codon is indicated by a black hexagon. (B) Total tocopherol was measured in leaves, mature green, and ripe fruits of M_4_ plants homozygous for the *folk-1* allele. The corresponding segregating individuals homozygous for the *FOLK* WT allele were used as control. Data represent the mean ±SD of five biological replicates. No significant differences were observed (Student’s *t*-test, *P*>0.05).

Due to sequence similarity between the tomato phosphatidate cytidylyltransferases proteins SlVTE5 and SlFOLK, and the lack of a complete functional characterization of the latter, the putative impact of SlFOLK on tocopherol metabolism was also explored. In this case, a TILLING-based molecular screening was applied to identify a loss-of-function mutation in *SlFOLK* using an EMS-mutagenized tomato collection. Among the identified mutants, one, named *folk-1*, displayed a G to A substitution disrupting the 3′ splicing site of intron 4. Sequence analyses of *folk-1* cDNA from homozygous mutant plants revealed that this lesion led to the use of a cryptic splicing site in intron 4, producing an mRNA that lacks exon 4 and contains a fragment of intron 4 (see Supplementary Fig. S4A at *JXB* online). This abnormally spliced transcript of *FOLK*, which is the only isoform detected in the mutant, contains an in-frame premature stop codon that presumably leads to a truncated protein (Fig. 4A; Supplementary Fig. S4A, B). mRNA harboring premature termination codons can be recognized by the RNA surveillance machinery as aberrant; these transcripts may be targeted by the nonsense-mediated decay pathway, being rapidly degraded (Filichkin *et al.*, 2015). Expression analysis by qPCR showed that the amount of the abnormal mRNA in *folk-1* corresponded to only 10% of the fully spliced transcript found in control plants (Supplementary Fig. S4C). Tocopherol levels and composition in plants homozygous for the *folk-1* allele were much the same as those in control plants, suggesting a small, if any, contribution of *SlFOLK* to tocopherol biosynthesis in both leaves and fruits (Fig. 4B; Supplementary Table S2). Having demonstrated the major role of *VTE5* in tomato tocopherol metabolism, we further performed a comprehensive phenotypic characterization of *SlVTE5*-RNAi lines.

### Down-regulation of *SlVTE5* boosted phytyl ester synthesis in leaves

**Fig. 5.**
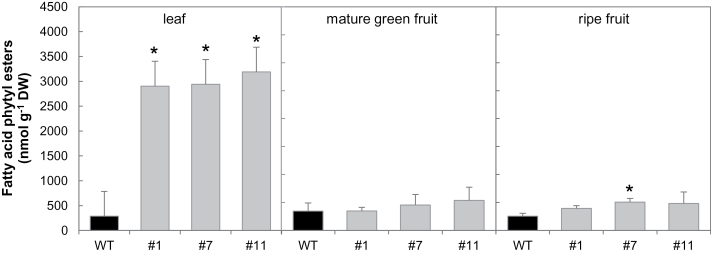
Total fatty acid phytyl ester (FAPE) content in *SlVTE5*-RNAi transgenic lines. Data represent the mean ±SD of at least three biological replicates. The asterisks denote significant differences between the wild-type (WT) and the transgenic lines (ANOVA/Dunnett’s test, *P*<0.05).

**Fig. 6.**
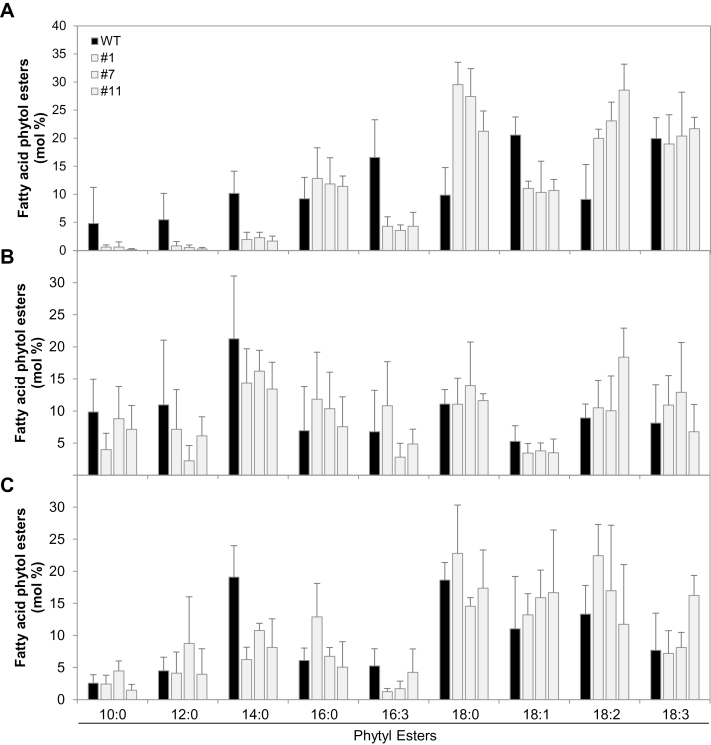
Molecular species composition of fatty acid phytyl esters (FAPEs) in *SlVTE5*-RNAi transgenic lines. FAPEs were measured in leaves (A), mature green (B), and ripe fruits (C). Data represent the mean ±SD of at least three biological replicates.

Free phytol can be esterified directly with fatty acids derived from activated acyl groups. FAPEs increase during stress-associated Chl degradation (e.g. nitrogen deprivation) and senescence (Gaude *et al.*, 2007; Lippold *et al.*, 2012). To address the question of whether the increased phytol levels affect FAPE contents in the *SlVTE5*-RNAi lines, the level of these compounds was measured by direct infusion Q-TOF MS/MS. Notably, *SlVTE5* knockdown resulted in a dramatic increase of FAPE content up to 10-fold in leaves. In contrast, fruits from *SlVTE5*-RNAi lines exhibited levels of FAPE identical to those of wild-type plants (Fig. 5). In addition to the total amount, FAPE composition was also highly affected in transgenic leaves (Fig. 6A). With the exception of palmitic (16:0) and linolenic (18:3) acids, analysis of the lipid profiles showed that the contribution of the different acyl chains was not proportional to the increment in total FAPE content observed in *SlVTE5*-RNAi lines. In particular, FAPEs containing oleic acid (18:1), hexadecatrienoic acid (16:3), and medium-chain fatty acids (10:0, 12:0, 14:0) exhibited a reduction in their relative content, while FAPEs containing stearic acid (18:0) and linoleic acid (18:2) became the predominant forms, increasing at least 2-fold in transgenic leaves compared with the wild-type. In *SlVTE5*-RNAi fruits, the FAPE composition at mature green and ripe stages remained almost unchanged (Fig. 6B, C).

### Chlorophyll content is not affected in *SlVTE5*-RNAi lines

**Fig. 7.**
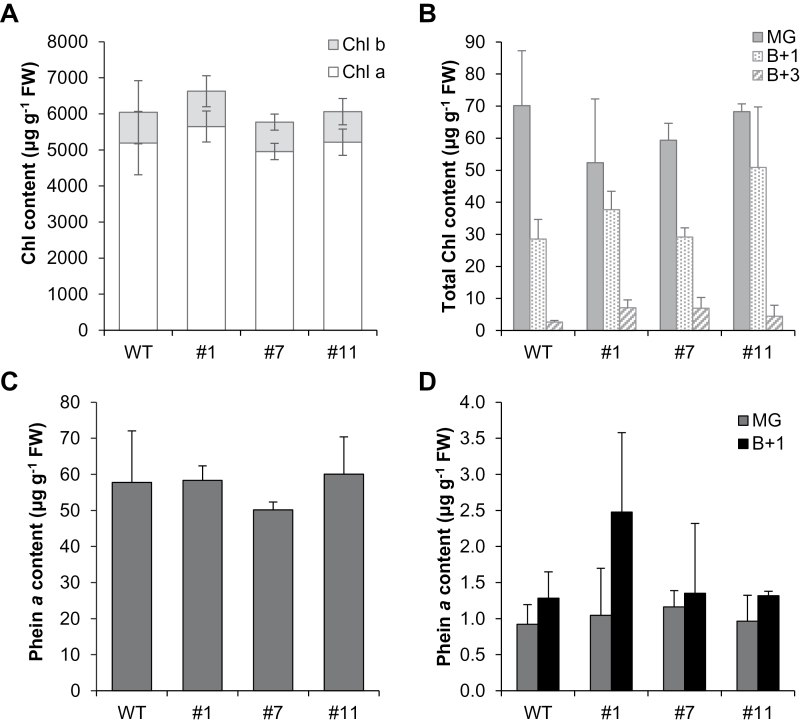
Chlorophyll (Chl) and pheophytin *a* (Phein *a*) content in *SlVTE5*-RNAi transgenic lines. (A, C) Quantification of Chl and Phein *a* in leaves. (B, D) Quantification of Chl and Phein *a* in fruits at mature green (MG), breaker+1 (B+1), and breaker+3 (B+3) stage. Data represent the mean ±SD of at least three biological replicates. No significant differences were observed (ANOVA/Dunnett’s test, *P*>0.05).

To examine whether *SlVTE5* knockdown affects Chl metabolism, we determined Chl *a*, Chl *b*, and pheophytin *a* (Phein *a*) levels in leaves and fruits at three different ripening stages by HPLC. The contents of these compounds were largely unaltered in both tested organs, suggesting that accumulation of phytol did not significantly affect Chl and Phein *a* levels in tomato (Fig. 7).

### *SlVTE5* knockdown alters prenyllipid metabolism in fruits

In addition to tocopherols, the plastidial antioxidant network includes a variety of prenyllipids derived from the MEP isoprenoid pathway with strong antioxidant properties, such as carotenoids, prenylquinones (PQ-9), and other tocochromanols (e.g. PC-8) (Nowicka *et al.*, 2013). To investigate whether tocopherol deficiency in *SlVTE5*-RNAi lines is compensated by any other non-enzymatic antioxidant mechanism, we performed a comprehensive profiling of prenyllipids (Table 1). For determination of prenylquinone and their derivative compounds, a targeted analysis of lipidomic profile obtained by the UHPLC-QTOF MS method was performed, whereas carotenoids were quantified by HPLC. In leaves and ripe fruits of the transgenic plants, depletion of tocopherol was accompanied by a decrease of α-tocopherolquinone, an oxidized intermediate of the tocopherol redox cycle; yet, the level of this metabolite remained unchanged in mature green fruits. Remarkably, the levels of the photosynthetic electron carrier phylloquinone (vitamin K), another product of phytyl diphosphate-dependent biosynthesis, did not change between *SlVTE5*-RNAi lines and the wild-type control.

**Table 1.** Changes in prenyllipid contents in leaves and fruits of *SlVTE5*-RNAi transgenic lines compared with the wild-type Data were normalized to sample DW and expressed relative to the wild-type (WT) in each tissue.

*Prenyllipids (relative amounts*)	WT	*SlVTE5*-RNAi		
		#1	#7	#11
Leaf				
α-TQ	1.00±0.18	**0.23±0.03**	**0.22±0.08**	**0.24±0.01**
PC-8	1.00±0.30	**0.52±0.19**	**0.54±0.19**	**0.36±0.07**
PQ-9	1.00±0.18	0.83±0.15	0.87±0.20	**0.63±0.12**
PQH_2_-9	1.00±0.66	1.27±0.59	1.02±0.43	0.86±0.37
PC-OH	1.00±0.21	**0.65±0.18**	0.72±0.24	**0.51±0.11**
PQ-OH	1.00±0.20	1.01±0.26	1.04±0.27	0.70±0.19
UQ-10	1.00±0.24	0.83±0.23	0.81±0.12	0.81±0.10
Phylloquinone	1.00±0.16	0.87±0.12	0.84±0.12	0.81±0.12
β-Carotene	1.00±0.07	0.89±0.04	0.91±0.02	0.91±0.07
Lutein	1.00±0.07	0.97±0.06	0.96±0.03	0.95±0.03
Violaxanthin/neoxanthin	1.00±0.06	0.97±0.06	1.00±0.02	0.99±0.06
Mature green fruit				
α-TQ	1.00±0.15	1.11±0.48	1.20±0.47	1.13±0.10
PC-8	1.00±0.15	**1.89±0.26**	**1.78±0.05**	**1.88±0.19**
PQ-9	1.00±0.15	**2.11±0.46**	**2.22±0.42**	**2.22±0.26**
PQH_2_-9	1.00±0.30	0.77±0.23	1.24±0.36	1.16±0.36
PC-OH	1.00±0.42	0.89±0.34	1.10±0.30	1.02±0.29
PQ-OH	1.00±0.26	1.31±0.49	**1.74±0.47**	1.52±0.32
UQ-10	1.00±0.13	**2.13±0.24**	**1.76±0.28**	**1.93±0.34**
Phylloquinone	1.00±0.27	1.23±0.18	1.25±0.32	1.32±0.16
β-Carotene	1.00±0.22	0.96±0.16	0.92±0.15	1.00±0.16
Lutein	1.00±0.22	0.98±0.10	1.09±0.11	**1.31±0.15**
Violaxanthin/neoxanthin	1.00±0.34	0.85±0.16	0.85±0.07	1.28±0.20
Ripe fruit				
α-TQ	1.00±0.25	**0.54±0.04**	**0.64±0.17**	**0.64±0.15**
PC-8	1.00±0.15	**2.29±0.34**	**1.67±0.20**	**2.07±0.50**
PQ-9	1.00±0.17	**2.40±0.54**	**2.24±0.20**	**1.76±0.26**
PQH_2_-9	1.00±0.29	1.55±0.39	1.77±0.55	1.92±1.06
PC-OH	1.00±0.27	1.26±0.51	1.35±0.46	0.99±0.42
PQ-OH	1.00±0.07	2.27±1.03	2.24±0.80	2.10±0.94
UQ-10	1.00±0.14	**2.40±0.38**	**1.96±0.53**	**2.13±0.24**
Phylloquinone	1.00±0.18	1.11±0.18	1.32±0.50	1.41±0.50
Phytoene	1.00±0.09	**0.68±0.11**	**0.72±0.19**	**0.62±0.10**
Phytofluene	1.00±0.14	**0.72±0.13**	**0.66±0.14**	**0.64±0.08**
Neurosporene	1.00±0.22	0.73±0.24	0.97±0.18	**0.60±0.20**
ζ-Carotene	1.00±0.20	**0.44±0.17**	**0.33±0.19**	**0.39±0.18**
Lycopene	1.00±0.06	**0.78±0.14**	**0.70±0.08**	**0.74±0.13**
β-Carotene	1.00±0.06	0.87±0.20	0.86±0.16	0.90±0.14
Lutein	1.00±0.13	1.07±0.13	1.02±0.09	1.19±0.25

Values are represented as means ±SD. Terms in bold indicate a statistically significant difference by ANOVA/Dunnett’s test (*P*<0.05).

α-TQ, α-tocopherolquinone; PQ-9, plastoquinone-9; PQH_2_-9, plastoquinol-9; PQ-OH, hydroxy-plastoquinone; PC-8, plastochromanol-8; PC-OH, hydroxy-plastochromanol; UQ-10, ubiquinone-10.

The presence of the reduced and oxidized forms of PQ-9 in wild-type tomato leaves has already been reported (Jones *et al.*, 2013), and our data showed that PC-8 is also detected in *S. lycopersicum*, in both leaves and fruits (see Supplementary Fig. S5 at *JXB* online). PC-8 was less abundant than tocopherols in wild-type tomato leaves, as described for other species (Kruk *et al.*, 2014). In fruits, however, the amount of these tocochromanols was similar (Fig. 2; Supplementary Fig. S5).

The comparison of prenyllipid profiles between wild-type and *SlVTE5*-knockdown leaves revealed that PQ-9 forms (PQ-9, PQH_2_-9, and PQ-OH) remained unchanged. In contrast, a reduction to 50% of PC-8 content was observed in *SlVTE5*-RNAi lines, which was accompanied by lower levels of its oxidation product, PC-OH (Table 1). In sharp contrast, the prenylquinone pool of transgenic fruits was significantly increased. PQ-9 levels were >2-fold higher in the *SlVTE5*-RNAi lines, although the levels of the reduced form PQH_2_-9 were unchanged. Intriguingly, the levels of the mitochondrial prenylquinone UQ-10 were also ~2-fold increased in *SlVTE5*-RNAi fruits. In addition, PC-8 accumulated up to 2-fold in both mature green and ripe fruits of the transgenic lines (Table 1). These results suggest that the pool of PQ-9 and PC-8 contributes to fulfilling an antioxidant function in the transgenic fruits.

Carotenoid contents in leaves and mature green fruits remained largely unaltered in the *SlVTE5*-RNAi lines (Table 1). Although no differences in visual appearance were identified (see Supplementary Fig. S3B at *JXB* online), at the ripe stage, silenced fruits exhibited 30% less lycopene than those of wild-type plants, which was also accompanied by an equivalent reduction in levels of lycopene precursors (phytoene, phytofluene, and ζ-carotene).

Considering the differences observed in prenyllipid profiles, the total antioxidant capacity of *SlVTE5*-RNAi and wild-type tomato plants was evaluated in non-polar extracts by the TEAC assay. Transgenic ripe fruits showed a reduction in TEAC values in lines #1 and #11 (see Supplementary Fig. S6 at *JXB* online). These results emphasize the role of tocopherol and/or carotenoids in antioxidant protection, since the increase in prenyllipid contents exhibited in the fruits of the *SlVTE5*-RNAi lines did not compensate the TEAC values up to the values observed in wild-type plants.

### *VTE5* deficiency affects the expression of tocopherol metabolism-related genes

**Fig. 8.**
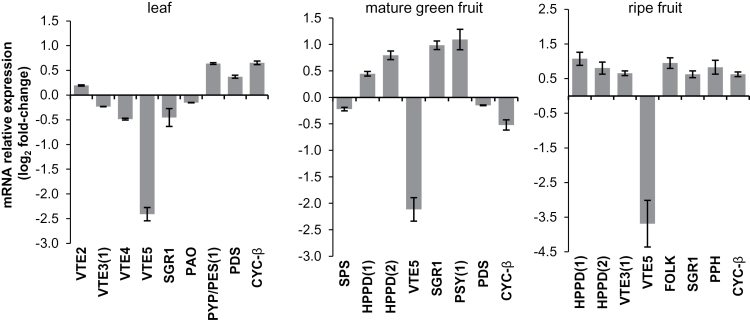
Changes in gene expression levels of some key isoprenoid metabolism-related genes resulting from *SlVTE5* down-regulation in both leaves and fruits.The amount of mRNA of the enzyme-encoding genes shown in Fig. 1 was quantified. Expression data are means ±SEM of three biological replicates of log2-fold changes compared with the corresponding organ of the wild-type control. Only genes that showed signiﬁcantly different mRNA levels in *SlVTE5*-knockdown lines are shown (permutation test, *P*<0.05). For simplicity, solely data from *SlVTE5*-RNAi#7 are represented. The complete data set is available in Supplementary Table S4 at *JXB* online.

The biochemical profile described above showed that *SlVTE5* knockdown results in an adjustment in prenyllipid and FAPE metabolism in an organ-specific manner. In order to understand whether these changes could be associated with differential gene expression regulation, mRNA levels of genes encoding proteins involved in MEP, shikimate, tocochromanol, carotenoid, and Chl metabolism (Quadrana *et al.*, 2013; Lira *et al.*, 2014; Almeida *et al.*, 2015), as well as in prenylquinone and FAPE synthesis were measured by qPCR (Fig. 1). Genes that showed signiﬁcantly different mRNA levels in at least two transgenic *SlVTE5*-RNAi lines and, when applicable, the third followed the same trend are shown in Fig. 8. The complete set of data is shown in Supplementary Table S4 at *JXB* online.

Genes of tocochromanol biosynthesis did not exhibit a consistent expression tendency in leaves of *SlVTE5*-RNAi lines. In fruit, the elevated expression of *HPPD(1*) and *HPPD(2*) is consistent with the increased prenylquinone content in silenced plants. Moreover, in transgenic ripe fruit, *VTE3(1*) up-regulation also coincided with the higher content of PC-8 and PQ-9. Intriguingly, the expression of the gene encoding the solanesyl diphosphate synthase (SPS), which catalyzes the production of the PQ-9 or PC-8 prenyl side chain, was decreased in transgenic mature green fruits, suggesting a negative feedback regulatory mechanism.

In leaves, an apparent reduction in the Chl degradation pathway was observed in *SlVTE5*-RNAi lines, as indicated by a down-regulation of *STAY-GREEN1* (*SGR1*) and *PHEOPHORBIDE A OXYGENASE* (*PAO*) expression. Although Chl contents remained invariant, the observed transcriptional down-regulation may reflect a response to phytol accumulation. This scenario contrasts with that observed in fruits where *SGR1* was up-regulated in transgenic plants compared with the wild-type. Additionally, ripe fruit of *SlVTE5*-RNAi lines showed higher mRNA levels of *PHEOPHYTINASE* (*PPH*).

Regarding carotenoid biosynthesis, only certain genes showed significant changes in their mRNA levels in leaves and mature green fruits of the *SlVTE5*-RNAi lines, although the biochemical profiles of these compounds remained unaltered when compared with the wild-type. In contrast, in ripe fruits, the increased levels of *CHROMOPLAST-SPECIFIC β-LYCOPENE CYCLASE* (*CYC-β*) transcripts could account for the reduction in lycopene and its immediate precursors verified in the transgenic lines.

Finally, the *PALE YELLOW PETAL 1* (*PYP1*) gene, the ortholog of Arabidopsis *PHYTYL ESTER SYNTHASE1* (*PES1*) (Ariizumi *et al.*, 2014), showed increased levels of transcripts in the leaves of the *SlVTE5*-RNAi lines, in agreement with the higher FAPE contents observed in these organs. Thus, the analysis of transcriptional profiles reinforces the specific regulation occurring in leaves and fruits.

### Measurements of carbohydrate metabolism, photosynthesis, and yield parameters suggest carbon export impairment in *SlVTE5*-knockdown plants

**Fig. 9.**
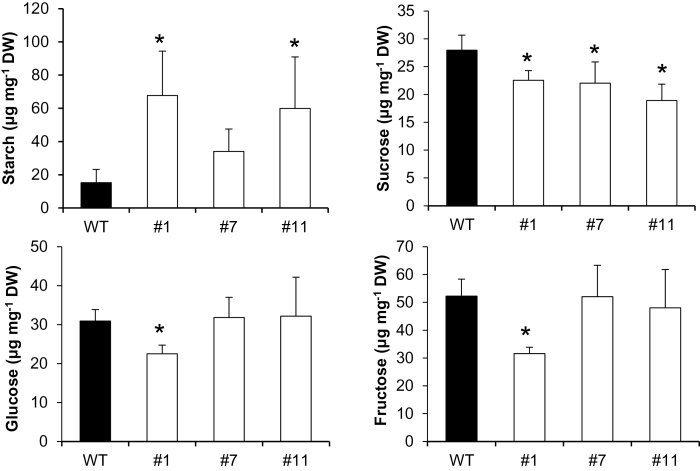
Starch and soluble sugar levels in source leaves in *SlVTE5*-RNAi transgenic lines. The first fully expanded leaves were harvested from 5-week-old plants in the middle of the light cycle. Starch is given in µg glucose equivalents. Data are means ±SD of five biological replicates. The asterisks denote significant differences between the wild-type (WT) and the transgenic lines (ANOVA/Dunnett’s test, *P*<0.05).

**Fig. 10.**
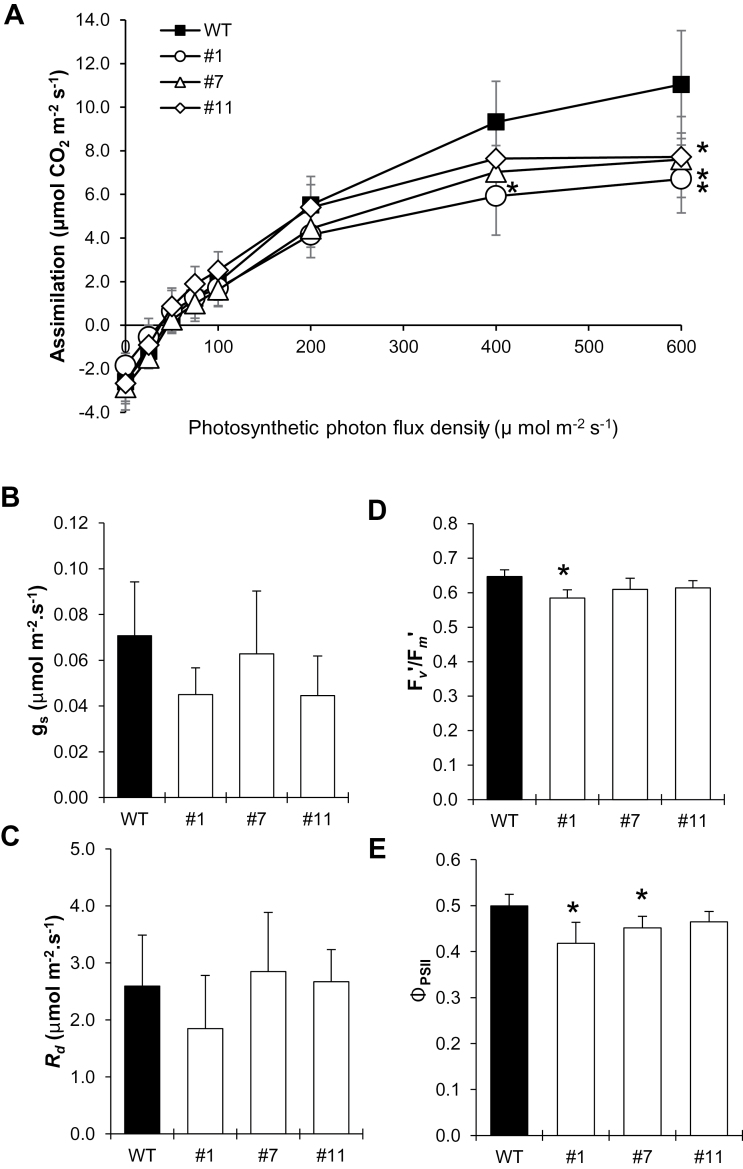
Gas-exchange and PSII efficiency parameters in *SlVTE5*-RNAi transgenic lines. (A) The response of carbon assimilation (*A*) to light intensity. (B) Leaf stomatal conductance (*g*_*s*_). (C) Leaf dark respiration (*R*_d_). (D) Light-adapted PSII maximum quantum efficiency (*F′*_v_/*F′*_m_). (E) PSII operating efficiency (Φ_PSII_). Data correspond to measurements in the first fully expanded leaf of 5-week-old plants and represent the means ±SD of five biological replicates. The asterisks denote significant differences between the wild-type (WT) and the transgenic lines (ANOVA/Dunnett’s test, *P*<0.05).

To evaluate whether the observed tocopherol deficiency in *SlVTE5*-RNAi lines affects carbon fixation and partitioning, the starch and soluble sugar content, photosynthetic performance, and yield parameters were assessed. Leaves of 5-week-old transgenic plants showed up to a 4.5-fold increase in starch content accompanied by a 20% decrease in sucrose levels compared with the wild-type control in the middle of the light period (Fig. 9). Concomitantly, the carbon assimilation rates were reduced in *SlVTE5*-RNAi lines, while the efficiency of PSII activity (Φ_PSII_) displayed moderate reduction (Fig. 10). The chloroplast ultrastructure was mostly preserved in *SlVTE5*-knockdown plants (see Supplementary Fig. S7 at *JXB* online).

In agreement with carotenoid profiling and the gene expression pattern, transgenic plants displayed a delay in fruit development and ripening as indicated by the frequency of red and green fruits, as well as the red fruit yield compared with the control genotype at harvest time (Table 2). Moreover, they displayed a yield penalty evidenced by a modest reduction in the harvest index. These results suggest that efficiency in photosynthate partitioning is compromised by the tocopherol deficiency in transgenic lines.

**Table 2.** Yield-associated traits of *SlVTE5*-RNAi transgenic lines

Trait	WT	*SlVTE5*-RNAi		
		#1	#7	#11
Number of total fruits	36.2±5.8	37.4±4.7	40.8±4.3	35.8±1.5
Frequency red fruits (%)	63.9±6.9	**49.6±7.8**	54.0±11.5	53.3±8.8
Frequency green fruits (%)	37.3±6.9	**50.4±7.8**	46.0±11.5	46.7±8.8
Vegetative plant weight (g FW)	30.0±8.3	34.3±8.0	39.4±6.9	34.0±7.3
Total yield per plant (g FW)	114.4±17.2	115.9±11.3	125.9±28.8	101.6±16.9
Harvest index	0.79±0.02	0.77±0.04	**0.76±0.02**	**0.75±0.03**
Red yield/aerial biomass ratio	0.57±0.08	**0.47±0.11**	**0.50±0.09**	0.52±0.15

Vegetative plant weight was determined by weighing only the vegetative tissue (after harvesting the fruits) without the root. Harvest index was calculated as the ratio between total fresh yield per plant (red and green fruit mass) and aerial biomass (total yield+vegetative plant weight).

Values indicate the mean ±SD of phenotypic values (*n*=5) determined for 15-week-old plants. Statistically significant differences between the wild-type (WT) control and transgenic lines are indicated in bold (Student’s *t*-test, *P*<0.05).

## Discussion

Several reports have dissected tocopherol biosynthesis, identified quantitative trait loci (QTLs) that determine VTE accumulation, and characterized regulatory mechanisms that control the tocopherol biosynthetic pathway (Almeida *et al.*, 2011; DellaPenna and Mène-Saffrané, 2011; Martinis et al., 2013, 2014; Quadrana *et al.*, 2013, 2014; Zhang *et al.*, 2013; Zhang *et al.*, 2014). One well-defined metabolic constraint is the availability of phytyl diphosphate precursor for tocopherol biosynthesis, which can be derived from *de novo* biosynthesis via the MEP pathway and from Chl phytol tail recycling (Ischebeck *et al.*, 2006; Quadrana *et al.*, 2013; Zhang *et al.*, 2013). The phytol hydrolysis of Chl is the primary source of prenyl chain for tocopherol biosynthesis in Arabidopsis seeds (Valentin *et al.*, 2006). However, functional analysis of the phytol salvage pathway has been limited to this species. In this study, we investigated the contribution of VTE5-mediated phytyl diphosphate synthesis for tocopherol production and its impact on plant physiology in tomato. The comprehensive metabolite profiling, expression analyses, and evaluation of photosynthetic parameters performed in tomato *SlVTE5*-knockdown plants allowed us to gain insights into the interactions between phytol recycling, and lipid and carbon metabolism, exposing distinct metabolic adjustment in source and sink organs.

Even with an active *de novo* synthesis of phytyl diphosphate, down-regulation of *SlVTE5* dramatically reduces tocopherol content in leaves and fruits in comparison with the wild-type genotype. These data demonstrated that in tomato, tocopherol production is mostly dependent on the Chl-linked pathway for phytyl diphosphate synthesis in both vegetative and reproductive organs. Furthermore, these findings suggest that *SlVTE5* is the main—if not the sole—contributor to VTE5 activity. The analysis of the tomato *folk-1* mutant suggested that *SlFOLK* is not involved in reactivation of free phytol.

Coincident with *SlVTE5* down-regulation, free phytol, the substrate of the phytol kinase reaction, accumulates in leaves of transgenic lines. Interestingly, the amount of free phytol accumulated corresponds to the decrease in tocopherol content, suggesting that a large proportion of phytol diphosphate derived from the phytol phosphorylation pathway is used for tocopherol biosynthesis in tomato. Furthermore, FAPEs are strongly increased in the mature leaves of *SlVTE5*-RNAi lines, although the Chl levels remain unchanged. FAPEs are plastogobule compounds that represent a class of stress-induced lipids in higher plants, which may act as plastidial transient sinks for the deposition of fatty acids and phytol (Gaude *et al.*, 2007). Since the phytol moiety of FAPEs is mostly derived from Chl degradation (Lippold *et al.*, 2012), our findings raise an intriguing issue concerning the origin of the phytol that sustains FAPE synthesis in *SlVTE5*-knockdown plants. One possible explanation may be that perturbations in phytol metabolism caused by the lack of VTE5 activity promote an increase in Chl turnover in transgenic lines. In leaves, the increased steady-state phytol levels were channeled to FAPE synthesis, while in fruits phytol remained as the free form. Alternatively, the origin of FAPE-associated phytol might be explained by the impairment in catabolism, which in plants involves the production of phytenoyl-CoA in chloroplasts that is further degraded by α-oxidation in peroxisomes and mitochondria, similar to that described in animals (Araújo *et al.*, 2011). Notably, levels of phylloquinone, another phytyl diphosphate chain-containing molecule, were unchanged in both leaves and fruits, indicating that the VTE5-dependent phytol pathway does not affect vitamin K synthesis in tomato. This result coincides with the previous observation of feeding experiments of Arabidopsis seedlings with radiolabeled phytol (Ischebeck *et al.*, 2006).

Even under permissive growing conditions, VTE5-tocopherol deficiency not only impacts the lipid profile but also carbon metabolism and photosynthesis, having consequences on the tomato plant physiology. *SlVTE5*-RNAi lines showed higher starch accumulation in mature leaves that correlated with a lower CO_2_ assimilation rate and the reduction in PSII operating efficiency. This scenario suggests carbohydrate-mediated feedback inhibition rather than a direct impact of tocopherol deficiency on photosynthetic capacity (Adams *et al.*, 2013; Asensi-Fabado *et al.*, 2014). Moreover, a subtle reduction in the number of harvestable fruits and the harvest index was observed. These results could be indicative of sugar export blockage from leaves towards sink organs in *SlVTE5*-knockdown tomato plants. Photoassimilate export impairment mediated by tocopherol deficiency has been reported in the literature, such as in potato *VTE1*-RNAi lines (Hofius *et al.*, 2004; Asensi-Fabado *et al.*, 2014) and *vte2 A. thaliana* during low-temperature adaptation (Maeda *et al.*, 2006, 2008), where carbon accumulation was verified in source leaves at the end of the light period. How tocopherol influences photoassimilate partitioning has not been precisely addressed yet. However, Song *et al.* (2010) provided robust genetic evidence that alterations in extra-plastidic lipid metabolism are upstream of the defect in photoassimilate export in VTE-deficient plants, which is mediated by fatty acid desaturases (FADs). In particular, it was reported that VTE depletion led to increased linoleic acid (18:2) content and a reduced level of linolenic acid (18:3). Consistent with this, one of the acyl groups that mostly contribute to the increase in the total FAPEs in the chloroplasts of the *SlVTE5*-RNAi lines was 18:2. Additionally, our lipidomic data revealed that the plastidial digalactosyldiacylglycerol (DGDG), which mainly consists of pairs including 18:3 species, was reduced in leaves of *SlVTE5*-RNAi lines compared with the wild-type (see Supplementary Table S5 at *JXB* online), resembling the lipid alterations previously reported (Maeda *et al.*, 2006, 2008). It has been proposed that changes in membrane lipid composition as a result of tocopherol deficiency might affect the properties of the secretory membrane systems (Maeda et al., 2008, 2014). Since tomato has been described as an apoplastic phloem loader (Muller *et al.*, 2014), we could speculate that the alteration of endomembrane vesicle formation affects the sucrose efflux mediated by SWEET proteins, which have been described as key players in phloem transport (Chen *et al.*, 2012; McCurdy and Hueros, 2014). Alternatively, the carbon export impairment observed in *SlVTE5*-knockdown tomato plants could be the result of the interaction between lipid and sugar metabolism by an as yet unidentified mechanism probably involving sugar sensing proteins, as proposed by Asensi-Fabado *et al.* (2014).

VTE5 deficiency triggered different metabolic responses in fruits compared with those described for leaves, reflecting the intrinsic physiological differences between organs and their corresponding plastids. First, fruits of *SlVTE5*-knockdown plants accumulated phytol in the free form rather than channeled into synthesis of FAPEs. This might be explained by the lowered availability of acyl donors inherent to fruit-specific lipid metabolism (Domínguez *et al.*, 2010). Secondly, the observation that the non-tocopherol prenylquinone pool, including PQ-9 and PC-8, is increased in fruits of *SlVTE5*-knockdown plants suggests that a regulatory compensation mechanism between the tocopherol and prenylquinone pathways exists in this organ. The ability of plastoquinol (PQH_2_-9), ubiquinol (UQH_2_-10), the reduced forms of PQ-9 and UQ-10, respectively, and PC-8 to scavenge ROS and inhibit lipid peroxidation has been demonstrated before (Kruk and Trebst, 2008; Nowicka *et al.*, 2013; Rastogi *et al.*, 2014). Moreover, PQ-9 and PC-8 have already been associated with inhibition of lipid peroxidation and ^1^O_2_ scavenging in VTE-deficient Arabidopsis *vte2* mutants (Mène-Saffrané *et al.*, 2010). Likewise, in tomato leaves, the reduction of PQ-9 content by the virus-induced gene silencing approach resulted in increased tocopherol and UQ-10 levels (Jones *et al.*, 2013). Interestingly, the favorable biochemical environment for the accumulation of PQ-9 and PC-8 is supported not only by reduced flux through the tocopherol competing pathway but also by the *HPPD* and *VTE3* up-regulation in this organ. Finally, *SlVTE5*-RNAi ripe fruits exhibited perturbations in the carotenoid pathway. The reduced amount of lycopene and its biosynthetic precursors can be explained by the higher transcript levels of *SGR1* found at the mature green and ripe stages. Besides having an important role in the regulation of plant Chl degradation and senescence (Hörtensteiner, 2009), SGR1 also regulates lycopene and β-carotene biosynthesis by direct interaction with PSY(1), thereby inhibiting its activity (Luo *et al.*, 2013). Simultaneously, the failure of co-ordinate transcriptional repression of the *CYC-β* gene in *SlVTE5*-knockdown ripe fruits could also account for the reduced abundance of acyclic carotenoids. The amounts of β-carotene and lutein in transgenic fruits were similar to those in the wild-type at the expense of the preceding carotenoids. Additionally, it has been demonstrated that the level of *PPH* transcripts decreases during tomato fruit ripening (Lira *et al.*, 2014); however, *SlVTE5*-RNAi ripe fruits displayed higher levels of *PPH* transcripts than the wild-type. Thus, the comprehensive analysis of the biochemical and transcriptional data together with the results of the yield experiment indicates that the ripe profiled transgenic fruits are indeed less ripe than wild-type fruits. The reduction in carbohydrate export described above might be in part responsible for the delay in fruit development and ripening, resulting in a reduced proportion of mature fruits at harvest time in *SlVTE5*-knockdown plants.

The results presented here clearly show that in tomato VTE biosynthesis is largely dependent on the salvage pathway for phytyl diphosphate synthesis rather than the *de novo* synthesis from the MEP pathway in both leaves and fruits. VTE5 deficiency affected lipid metabolism, evidenced by the abundance and composition of FAPEs in leaves and prenylquinones in fruits. Together, these results exposed the complexities of the metabolic regulation that emerge from the isoprenoid pathway network, which involves a tight control between precursor supply and utilization highly dependent on the plastid type. Moreover, our data highlighted the cross-talk between lipid and carbon metabolism mediated by tocopherol that resulted in the impairment of carbon export in *SlVTE5*-knockdown tomato plants compromising fruit development and ripening.

## Supplementary data

Supplementary data are available at *JXB* online.

Table S1. Primers used for each experiment.

Table S2. Tocopherol content and composition of *SlVTE5*-RNAi transgenic lines and the *folk-1* mutant.

Table S3. Moles of prenyllipids found in *SlVTE5*-RNAi transgenic lines.

Table S4. Transcriptional profile of genes encoding isoprenoid metabolism-related enzymes.

Table S5. Changes in fatty acid-derived lipids in leaves of *SlVTE5*-RNAi transgenic lines compared with the wild-type.

Fig. S1. Phylogenetic analysis of VTE5 and FOLK proteins.

Fig. S2. Expression of *SlVTE5* and *SlFOLK*.

Fig. S3. Fruit phenotype of *SlVTE5*-RNAi transgenic lines.

Fig. S4. Analysis of the *folk-1* mutation.

Fig. S5. Plastoquinone (PQ-9) and plastochromanol (PC-8) levels in *SlVTE5*-RNAi transgenic lines.

Fig. S6. Trolox equivalent antioxidant capacity (TEAC) in leaves and fruits of *SlVTE5*-RNAi transgenic lines.

Fig. S7. Chloroplast ultrastructure resulting from *SlVTE5* down-regulation.

Supplementary Data

## References

[CIT0001] Abbasi AR Hajirezaei M Hofius D Sonnewald U Voll LM 2007 Specific roles of α- and γ-tocopherol in abiotic stress responses of transgenic tobacco. Plant Physiology 143, 1720–1738. 17293434 10.1104/pp.106.094771 PMC1851823

[CIT0002] Adams WW Muller O Cohu CM Demmig-Adams B 2013 May photoinhibition be a consequence, rather than a cause, of limited plant productivity? Photosynthesis Research 117, 31–44. 23695654 10.1007/s11120-013-9849-7

[CIT0003] Almeida J Asís R Molineri VN Sestari I Lira BS Carrari F Peres LE Rossi M 2015 Fruits from ripening impaired, chlorophyll degraded and jasmonate insensitive tomato mutants have altered tocopherol content and composition. Phytochemistry 111, 72–83. 25432273 10.1016/j.phytochem.2014.11.007

[CIT0004] Almeida J Quadrana L Asís R 2011 Genetic dissection of vitamin E biosynthesis in tomato. Journal of Experimental Botany 62, 3781–3798. 21527625 10.1093/jxb/err055 PMC3134339

[CIT0005] Amaral LIV Gaspar M Costa PF Aidar MPM Buckeridge MS 2008 Novo método enzimático rápido e sensível de extração e dosagem de amido em materiais vegetais. Hoehnea 34, 425–431.

[CIT0006] Araújo WL Ishizaki K Nunes-Nesi A 2011 Analysis of a range of catabolic mutants provides evidence that phytanoyl-coenzyme A does not act as a substrate of the electron-transfer flavoprotein/electron-transfer flavoprotein, ubiquinone oxidoreductase complex in Arabidopsis during dark-induced senescence. Plant Physiology 157, 55–69. 21788362 10.1104/pp.111.182188 PMC3221279

[CIT0007] Ariizumi T Kishimoto S Kakami R 2014 Identification of the carotenoid modifying gene PALE YELLOW PETAL 1 as an essential factor in xanthophyll esterification and yellow flower pigmentation in tomato (*Solanum lycopersicum*). The Plant Journal 79, 453–465. 24888879 10.1111/tpj.12570

[CIT0008] Asensi-Fabado MA Ammon A Sonnewald U Munné-Bosch S Voll LM 2014 Tocopherol deficiency reduces sucrose export from salt-stressed potato leaves independently of oxidative stress and symplastic obstruction by callose. Journal of Experimental Botany 66, 957–971. 25428995 10.1093/jxb/eru453 PMC4321552

[CIT0009] Botella-Pavía P Besumbes O Phillips MA Carretero-Paulet L Boronat A Rodriguez-Concepcion M 2004 Regulation of carotenoid biosynthesis in plants, evidence for a key role of hydroxymethylbutenyl diphosphate reductase in controlling the supply of plastidial isoprenoid precursors. The Plant Journal 40, 188–199. 15447646 10.1111/j.1365-313X.2004.02198.x

[CIT0010] Chen LQ Qu XQ Hou BH Sosso D Osorio S Fernie AR Frommer WB 2012 Sucrose efflux mediated by SWEET proteins as a key step for phloem transport. Science 335, 207–211. 22157085 10.1126/science.1213351

[CIT0011] Chun J Lee J Ye L Exler J Eitenmiller RR 2006 Tocopherol and tocotrienol contents of raw and processed fruits and vegetables in the United States diet. Journal of Food Composition and Analysis 19, 196–204.

[CIT0012] DellaPenna D Mène-Saffrané L 2011 Vitamin E. Advances in Botanical Research 59, 179–227.

[CIT0013] DellaPenna D Pogson BJ 2006 Vitamin synthesis in plants: tocopherols and carotenoids. Annual Review of Plant Biology 57, 711–738. 10.1146/annurev.arplant.56.032604.144301 16669779

[CIT0014] Demmig-Adams B Stewart JJ Adams WW 2014 Multiple feedbacks between chloroplast and whole plant in the context of plant adaptation and acclimation to the environment. Philosophical Transactions of the Royal Society B: Biological Sciences 369, 20130244. 10.1098/rstb.2013.0244 PMC3949402 24591724

[CIT0015] De Souza AP Arundale RA Dohleman FG Long SP Buckeridge MS 2013 Will the exceptional productivity of *Miscanthus×giganteus* increase further under rising atmospheric CO_2_? Agricultural and Forest Meteorology 171–172, 82–92.

[CIT0016] Di Mascio P Devasagayam TP Kaiser S Sies H 1990 Carotenoids, tocopherols and thiols as biological singlet molecular oxygen quenchers. Biochemical Society Transactions 18, 1054–1056. 2088803 10.1042/bst0181054

[CIT0017] Di Rienzo JA 2009 Statistical software for the analysis of experiments of functional genomics. RDNDA, Argentina http://sites.google.com/site/fgStatistics/.

[CIT0018] Domínguez T Hernández ML Pennycooke JC Jiménez P Martínez-Rivas JM Sanz C Stockinger EJ Sánchez-Serrano JJ Sanmartín M 2010 Increasing ω-3 desaturase expression in tomato results in altered aroma profile and enhanced resistance to cold stress. Plant Physiology 153, 655–665. 20382895 10.1104/pp.110.154815 PMC2879794

[CIT0019] Edreva A 2005 Generation and scavenging of reactive oxygen species in chloroplasts, a submolecular approach. Agriculture, Ecosystems and Environment 106, 119–133.

[CIT0020] Eugeni-Piller L Glauser G Kessler F Besagni C 2014 Role of plastoglobules in metabolite repair in the tocopherol redox cycle. Frontiers in Plant Science 5, 298. 25018761 10.3389/fpls.2014.00298 PMC4071476

[CIT0021] Filichkin S Priest HD Megraw M Mockler TC 2015 Alternative splicing in plants: directing traffic at the crossroads of adaptation and environmental stress. Current Opinion in Plant Biology 24, 125–135. 25835141 10.1016/j.pbi.2015.02.008

[CIT0022] Fitzpatrick AH Bhandari J Crowell DN 2011 Farnesol kinase is involved in farnesol metabolism, ABA signaling and flower development in *Arabidopsis* . The Plant Journal 66, 1078–1088. 21395888 10.1111/j.1365-313X.2011.04572.x

[CIT0023] Fitzpatrick TB Basset GJC Borel P 2012 Vitamin deficiencies in humans: can plant science help? The Plant Cell 24, 395–414. 22374394 10.1105/tpc.111.093120 PMC3315223

[CIT0024] Foyer CH Noctor G 2005 Redox homeostasis and antioxidant signaling: a metabolic interface between stress perception and physiological responses. The Plant Cell 17, 1866–1875. 15987996 10.1105/tpc.105.033589 PMC1167537

[CIT0025] Fukuzawa K Matsuura K Tokumura A Suzuki A Terao J 1997 Kinetics and dynamics of singlet oxygen scavenging by alpha-tocopherol in phospholipid model membranes. Free Radical Biology and Medicine 22, 923–930. 9119263 10.1016/s0891-5849(96)00485-6

[CIT0026] Gaude N Bréhélin C Tischendorf G Kessler F Dörmann P 2007 Nitrogen deficiency in *Arabidopsis* affects galactolipid composition and gene expression and results in accumulation of fatty acid phytyl esters. The Plant Journal 49, 729–739. 17270009 10.1111/j.1365-313X.2006.02992.x

[CIT0027] Genty B Briantais JM Baker NR 1989 The relationship between quantum yield of photosynthetic electron transport and quenching of chlorophyll fluorescence. Biochimica et Biophysica Acta 990, 87–92.

[CIT0028] Grusak MA DellaPenna D 1999 Improving the nutrient composition of plants to enhance human nutrition and health. Annual Review of Plant Physiology and Plant Molecular Biology 50, 133–161. 10.1146/annurev.arplant.50.1.133 15012206

[CIT0029] Gupta P Sreelakshmi Y Sharma R 2015 A rapid and sensitive method for determination of carotenoids in plant tissues by high performance liquid chromatography. Plant Methods 11, 5. 25688283 10.1186/s13007-015-0051-0 PMC4329677

[CIT0030] Havaux M Eymery F Porfirova S Rey P Dörmann P 2005 Vitamin E protects against photoinhibition and photooxidative stress in *Arabidopsis thaliana* . The Plant Cell 17, 3451–3469. 16258032 10.1105/tpc.105.037036 PMC1315381

[CIT0031] Hofius D Hajirezaei MR Geiger M Tschiersch H Melzer M Sonnewald U 2004 RNAi-mediated tocopherol deficiency impairs photoassimilate export in transgenic potato plants. Plant Physiology 135, 1256–1268. 15247386 10.1104/pp.104.043927 PMC519045

[CIT0032] Hörtensteiner S 2009 Stay-green regulates chlorophyll and chlorophyll-binding protein degradation during senescence. Trends in Plant Sciences 14, 155–162. 10.1016/j.tplants.2009.01.002 19237309

[CIT0033] Ischebeck T Zbierzak AM Kanwischer M Dörmann P 2006 A salvage pathway for phytol metabolism in *Arabidopsis* . Journal of Biological Chemistry 281, 2470–2477. 16306049 10.1074/jbc.M509222200

[CIT0034] Jones MO Perez-Fons L Robertson FP Bramley PM Fraser PD 2013 Functional characterization of long-chain prenyl diphosphate synthases from tomato. Biochemical Journal 449, 729–740. 23126257 10.1042/BJ20120988

[CIT0035] Just D Garcia V Fernandez L 2013 Micro-Tom mutants for functional analysis of target genes and discovery of new alleles in tomato. Plant Biotechnology 30, 225–231.

[CIT0036] Kaiser S Di Mascio P Murphy ME Sies H 1990 Physical and chemical scavenging of singlet molecular oxygen by tocopherols. Archives of Biochemistry and Biophysics 277, 101–108. 2306113 10.1016/0003-9861(90)90556-e

[CIT0037] Kamal-Eldin A Appelqvist LA 1996 The chemistry and antioxidant properties of tocopherols and tocotrienols. Lipids 31, 671–701. 8827691 10.1007/BF02522884

[CIT0038] Karimi M Inzé D Depicker A 2002 GATEWAY vectors for Agrobacterium-mediated plant transformation. Trends in Plant Science 7, 193–195. 11992820 10.1016/s1360-1385(02)02251-3

[CIT0039] Kruk J Szymańska R Cela J Munné-Bosch S 2014 Plastochromanol-8: fifty years of research. Phytochemistry 108, 9–16. 25308762 10.1016/j.phytochem.2014.09.011

[CIT0040] Kruk J Trebst A 2008 Plastoquinol as a singlet oxygen scavenger in photosystem II. Biochimica et Biophysica Acta 1777, 154–162. 18005659 10.1016/j.bbabio.2007.10.008

[CIT0041] Langmeier M Ginsburg S Matile P 1993 Chlorophyll breakdown in senescent leaves: demonstration of Mg-dechelatase activity. Physiologia Plantarum 89, 347–353.

[CIT0042] Lippold F vom Dorp K Abraham M 2012 Fatty acid phytyl ester synthesis in chloroplasts of *Arabidopsis* . The Plant Cell 24, 2001–2014. 22623494 10.1105/tpc.112.095588 PMC3442583

[CIT0043] Lira B Setta N Rosado D Almeida J Freschi L Rossi M 2014 Plant degreening: evolution and expression of tomato (*Solanum lycopersicum*) dephytylation enzymes. Gene 546, 359–366. 24865932 10.1016/j.gene.2014.05.051

[CIT0044] Loyola J Verdugo I González E Casaretto JA Ruiz-Lara S 2012 Plastidic isoprenoid biosynthesis in tomato: physiological and molecular analysis in genotypes resistant and sensitive to drought stress. Plant Biology 14, 149–156. 21974688 10.1111/j.1438-8677.2011.00465.x

[CIT0045] Luo Z Zhang J Li J Yang C Wang T Ouyang B Li H Giovannoni J Ye Z 2013 A STAY-GREEN protein SlSGR1 regulates lycopene and β-carotene accumulation by interacting directly with SlPSY1 during ripening processes in tomato. New Phytologist 198, 442–452. 23406468 10.1111/nph.12175

[CIT0046] Maeda H Sage TL Isaac G Welti R Dellapenna D 2008 Tocopherols modulate extraplastidic polyunsaturated fatty acid metabolism in *Arabidopsis* at low temperature. The Plant Cell 20, 452–470. 18314499 10.1105/tpc.107.054718 PMC2276453

[CIT0047] Maeda H Song W Sage TL DellaPenna D 2006 Tocopherols play a crucial role in low-temperature adaptation and phloem loading in *Arabidopsis* . The Plant Cell 18, 2710–2732. 17012603 10.1105/tpc.105.039404 PMC1626601

[CIT0048] Maeda H Song W Sage T DellaPenna D 2014 Role of callose synthases in transfer cell wall development in tocopherol deficient mutants. Frontiers in Plant Science 5, 46. 24600460 10.3389/fpls.2014.00046 PMC3928550

[CIT0049] Martinis J Glauser G Valimareanu S Kessler F 2013 A chloroplast ABC1-like kinase regulates vitamin E metabolism in *Arabidopsis thaliana* . Plant Physiology 162, 652–662. 23632854 10.1104/pp.113.218644 PMC3668060

[CIT0050] Martinis J Glauser G Valimareanu S Stettler M Zeeman S Yamamoto H Shikanai T Kessler F 2014 ABC1K1/PGR6 kinase: a regulatory link between photosynthetic activity and chloroplast metabolism. The Plant Journal 77, 268–283. 10.1111/tpj.12385 24267661

[CIT0051] McCurdy DW Hueros G 2014 Transfer cells. Frontiers in Plant Science 5, 672. 25505481 10.3389/fpls.2014.00672 PMC4245004

[CIT0052] Mène-Saffrané L Jones AD DellaPenna D 2010 Plastochromanol-8 and tocopherols are essential lipid-soluble antioxidants during seed desiccation and quiescence in *Arabidopsis* . Proceedings of the National Academy of Sciences, USA 107, 17815–17820. 10.1073/pnas.1006971107 PMC2955118 20837525

[CIT0053] Miret JA Munné-Bosch S 2015 Redox signaling and stress tolerance in plants: a focus on vitamin E. Annals of the New York Academy of Sciences 1340, 29–38. 25586886 10.1111/nyas.12639

[CIT0054] Muller O Cohu CM Stewart JJ Protheroe JA Demmig-Adams B Adams WW 2014 Association between photosynthesis and contrasting features of minor veins in leaves of summer annuals loading phloem via symplastic versus apoplastic routes. Physiologia Plantarum 152, 174–183. 24450755 10.1111/ppl.12155

[CIT0055] Munné-Bosch S 2005 The role of a-tocopherol in plant stress tolerance. Journal of Plant Physiology 162, 743–748. 16008098 10.1016/j.jplph.2005.04.022

[CIT0056] Ng PC Henikoff S 2003 SIFT: predicting amino acid changes that affect protein function. Nucleic Acids Research 31, 3812–3814. 12824425 10.1093/nar/gkg509 PMC168916

[CIT0057] Nowicka B Gruszka J Kruk J 2013 Function of plastochromanol and other biological prenyllipids in the inhibition of lipid peroxidation—a comparative study in model systems. Biochimica et Biophysica Acta 1828, 233–240. 22959712 10.1016/j.bbamem.2012.08.018

[CIT0058] Okabe Y Asamizu E Saito T Matsukura C Ariizumi T Brès C Rothan C Mizoguchi T Ezura H 2011 Tomato TILLING technology: development of a reverse genetics tool for the efﬁcient isolation of mutants from Micro-Tom mutant libraries. Plant and Cell Physiology 52, 1994–2005. 21965606 10.1093/pcp/pcr134 PMC3212723

[CIT0059] Pfaffl MW Horgan GW Dempfle L 2002 Relative expression software tool (REST) for group-wise comparison and statistical analysis of relative expression results in real-time PCR. Nucleic Acids Research 30, e36. 11972351 10.1093/nar/30.9.e36 PMC113859

[CIT0060] Pino LE Lombardi-Crestana S Azevedo MS Scotton DC Borgo L Quecini V Figueira A Peres LE 2010 The Rg1 allele as a valuable tool for genetic transformation of the tomato ‘Micro-Tom’ model system. Plant Methods 6, 23. 20929550 10.1186/1746-4811-6-23 PMC2958934

[CIT0061] Quadrana L Almeida J Asís R 2014 Natural occurring epialleles determine vitamin E accumulation in tomato fruits. Nature Communications 5, 3027. 10.1038/ncomms5027 24967512

[CIT0062] Quadrana L Almeida J Otaiza SN 2013 Transcriptional regulation of tocopherol biosynthesis in tomato. Plant Molecular Biology 81, 309–325. 23247837 10.1007/s11103-012-0001-4

[CIT0063] Rastogi A Yadav DK Szymaska R Kruk J Sedlarova M Pospísil P 2014 Singlet oxygen scavenging activity of tocopherol and plastochromanol in *Arabidopsis thaliana*: relevance to photooxidative stress. Plant, Cell and Environment 37, 392–401. 10.1111/pce.12161 23848570

[CIT0064] Re R Pellegrini N Proteggente A Pannala A Yang A Rice-Evans C 1999 Antioxidant activity applying an improved ABTS radical cation decolorization assay. Free Radical Biology and Medicine 26, 1231–1237. 10381194 10.1016/s0891-5849(98)00315-3

[CIT0065] Reynolds ES 1963 The use of lead citrate at high pH as an electron-opaque stain in electron microscopy. Journal of Cell Biology 17, 208–212. 13986422 10.1083/jcb.17.1.208 PMC2106263

[CIT0066] Sattler SE Gilliland LU Magallanes-Lundback M Pollard M Dellapenna D 2004 Vitamin E is essential for seed longevity and for preventing lipid peroxidation during germination. The Plant Cell 16, 1419–1432. 15155886 10.1105/tpc.021360 PMC490036

[CIT0067] Schelbert S Aubry S Burla B Agne B Kessler F Krupinska K Hörtensteiner S 2009 Pheophytin pheophorbide hydrolase (pheophytinase) is involved in chlorophyll breakdown during leaf senescence in *Arabidopsis* . The Plant Cell 21, 767–785. 19304936 10.1105/tpc.108.064089 PMC2671698

[CIT0068] Serbinova E Kagan V Han D Packer L 1991 Free radical recycling and intramembrane mobility in the antioxidant properties of alpha-tocopherol and alpha-tocotrienol. Free Radical Biology and Medicine 10, 263–275. 1649783 10.1016/0891-5849(91)90033-y

[CIT0069] Seymour GB Østergaard L Chapman NH Knapp S Martin C 2013 Fruit development and ripening. Annual Review of Plant Biology 64, 219–241. 10.1146/annurev-arplant-050312-120057 23394500

[CIT0070] Song W Maeda H Dellapenna D 2010 Mutations of the ER to plastid lipid transporters TGD1, 2, 3 and 4 and the ER oleate desaturase FAD2 suppress the low temperature-induced phenotype of *Arabidopsis* tocopherol-deficient mutant *vte2* . The Plant Journal 62, 1004–1018. 20345604 10.1111/j.1365-313X.2010.04212.x

[CIT0071] Tamura K Dudley J Nei M Kumar S 2007 MEGA4: Molecular Evolutionary Genetics Analysis (MEGA) software version 4.0. Molecular Biology and Evolution 24, 1596–1599. 17488738 10.1093/molbev/msm092

[CIT0072] Taylor NE Greene EA 2003 PARSESNP: a tool for the analysis of nucleotide polymorphisms. Nucleic Acids Research 31, 3808–3811. 12824424 10.1093/nar/gkg574 PMC168980

[CIT0073] Traber MG Atkinson J 2008 Vitamin E, antioxidant and nothing more. Free Radical Biology and Medicine 43, 4–15. 17561088 10.1016/j.freeradbiomed.2007.03.024 PMC2040110

[CIT0074] Till BJ Reynolds SH Greene EA 2003 Large-scale discovery of induced point mutations with high-throughput TILLING. Genome Research 13, 524–530. 12618384 10.1101/gr.977903 PMC430291

[CIT0075] Triantaphylidès C Havaux M 2009 Singlet oxygen in plants: production, detoxification and signaling. Trends in Plant Science 14, 219–228. 19303348 10.1016/j.tplants.2009.01.008

[CIT0076] Valentin HE Lincoln K Moshiri F 2006 The *Arabidopsis* vitamin E pathway gene5-1 mutant reveals a critical role for phytol kinase in seed tocopherol biosynthesis. The Plant Cell 18, 212–224. 16361393 10.1105/tpc.105.037077 PMC1323494

[CIT0077] Vicente MH Zsögön A Sá AFL Ribeiro RV Peres LEP 2015 Semi-determinate growth habit adjusts the vegetative-to-reproductive balance and increases productivity and water-use efficiency in tomato (*Solanum lycopersicum*). Journal of Plant Physiology 177, 11–19. 25659332 10.1016/j.jplph.2015.01.003

[CIT0078] vom Dorp K Hölzl G Plohmann C Eisenhut M Abraham M Weber AP Hanson AD Dörmann P 2015 Remobilization of phytol from chlorophyll degradation is essential for tocopherol synthesis and growth of Arabidopsis. The Plant Cell 27, 2846–2849 26452599 10.1105/tpc.15.00395 PMC4682318

[CIT0079] Watson ML 1958 Staining of tissue sections for electron microscopy with heavy metals. Journal of Biophysical and Biochemical Cytology 4, 475–478. 13563554 10.1083/jcb.4.4.475 PMC2224499

[CIT0080] Welti R Li W Li M Sang Y Biesiada H Zhou HE Rajashekar CB Williams TD Wang X 2002 Profiling membrane lipids in plant stress responses. Role of phospholipase D alpha in freezing-induced lipid changes in *Arabidopsis* . Journal of Biological Chemistry 277, 31994–32002. 12077151 10.1074/jbc.M205375200

[CIT0081] Yang W Cahoon RE Hunter SC Zhang C Han J Borgschulte T Cahoon EB 2011 Vitamin E biosynthesis, functional characterization of the monocot homogentisate geranylgeranyl transferase. The Plant Journal 65, 206–217. 21223386 10.1111/j.1365-313X.2010.04417.x

[CIT0082] Zbierzak AM Kanwischer M Wille C 2010 Intersection of the tocopherol and plastoquinol metabolic pathways at the plastoglobule. Biochemical Journal 425, 389–399. 19843012 10.1042/BJ20090704

[CIT0083] Zhang C Cahoon RE Hunter SC Chen M Han J Cahoon EB 2013 Genetic and biochemical basis for alternative routes of tocotrienol biosynthesis for enhanced vitamin E antioxidant production. The Plant Journal 73, 628–639. 23137278 10.1111/tpj.12067

[CIT0084] Zhang W Liu T Ren G Hörtensteiner S Zhou Y Cahoon EB Zhang C 2014 Chlorophyll degradation: the tocopherol biosynthesis related phytol hydrolase in *Arabidopsis* seeds is still missing. Plant Physiology 166, 70–79. 25059706 10.1104/pp.114.243709 PMC4149732

