# Supplementary material for: Down-regulation of tomato PHYTOL KINASE strongly impairs tocopherol biosynthesis and affects prenyllipid metabolism in an organ-specific manner
Source: J Exp Bot. 2015 Nov 23;67(3):919–34. doi: 10.1093/jxb/erv504 (PMC4737080; doi:10.1093/jxb/erv504)
Supplement: Supplementary Data [file supp_67_3_919__index.html]

Down-regulation of tomato PHYTOL KINASE strongly impairs tocopherol biosynthesis and affects prenyllipid metabolism in an organ-specific manner — Down-regulation of tomato PHYTOL KINASE strongly impairs tocopherol biosynthesis and affects prenyllipid metabolism in an organ-specific manner — Supplementary Data 

# Down-regulation of tomato *PHYTOL KINASE* strongly impairs tocopherol biosynthesis and affects prenyllipid metabolism in an organ-specific manner

## Supplementary Data

Data files

- supplementary\_tables\_S1\_S5\_Figures\_S1\_S7.pdf - Supplementary Data
