# Supplementary material for: Down-regulation of tomato PHYTOL KINASE strongly impairs tocopherol biosynthesis and affects prenyllipid metabolism in an organ-specific manner
Source: J Exp Bot. 2015 Nov 23;67(3):919–34. doi: 10.1093/jxb/erv504 (PMC4737080; doi:10.1093/jxb/erv504)
Supplement: Supplementary Data [file supp_erv504_supplementary_tables_S1_S5_Figures_S1_S7.pdf]

Supplementary Table S1. Primers used for each experiment.

Experiment	Primer name[a]	Primer Sequence (5'-3') Foward	Primer Sequence (5'-3') Reverse
qPCR	DXS(1)	CAGGACTGGTGTGGTTTCAG	GGGATAGTTCACAGTGTCC
	GGPS(2)	GTTGATTCATGGGGTCAAGC	CAAATCGCCTTTTCAGCTACG
	GGDR	CAGAGACGCTCGCTAAGG	GCTTCAGAGTCTGTCCGATATC
	HPPD(1)	CCAGGGCAGGGGATATACTG	CTCCTTCCTCGTTTTTCAGC
	HPPD(2)	CCAGGCGTGTGAAGAATTG	CGATCTAAACAGCTCAGAG
	VTE2	CAATTCCAGTTCCTGCTGAG	CCTCCAACATGCTCTTGCGTG
	VTE3(1)	CTTGACCAATCTCCTCATC	GCACGCCTTTCCTCCAGG
	VTE3(2)	GCTAAGGCTAGGCAGAAGGAG	CAGGCAACCCCACCTATGG
	VTE1	CGAACTCCTCATAGCGGGTATC	CACGCCAGTAAACCGAGGC
	VTE4	CAGATCATCGTGCTGCTCAG	CCTCTCTGCTTGTACAGGAC
	VTE5	CGTATCAGGACGGGCTCGC	TCACCACCACACATCATTGCTAATG
	FOLK	CTATGAGCCGATTTGGAGACC	GAACCTCCTGCCAACAATGTC
	SGR1	GCAAAGAACTCCCTGTGGTT	CCCACCAGAAGAAGATGAGG
	CHLG	CCAATTCCTTCAGGTGCGGT	CCCACCAAGGCAAGCTGATA
	PPH	TATGGAGGGAGCAAGTACGC	TGGAGGGCAGAGGAAAAGTAC
	PAO	TCAGAAGTGGGTGATATGGA	TATCCCCGTCATACACCTTA
	HST	GCTGCTAACTTGGTGCTC	GATCCTAGCACAGTCCCACG
	SPS	GTGGTTGCGGATGACCTACTTA	CTTCTGTGATTTGTGGTGAGTTCC
	PSY(1)	CGATGGTGCTTTGTCCGATAC	CTCATCAACCCAACCGTACC
	PSY(2)	GCATCACACATAACTCCACAAGC	CGCATTCCTTCAACCATATCTCTG
	PDS	CGTTCCGTGCTTCTCCGC	CTAGAACATCCCTTGCCTCCAG
	LCYβ	GCACCCACATCAAAGCCAGAG	GCCACATGGAGAGTGGTGAAG
	CYCβ	TTGACTTAGAACCTCGTTATTGG	AACAGTTCCCTTTGTCATTATCT
	PYP/PES(1)	ACAGGACACAACTCCAACC	TAACCATCGCCATCTTCAGTG
	PES(2)	CGAAGAGAGGGAAAAATGCCTGTG	GCTGCCATCCTGACAAATTCAGAC
	CAC	CCTCCGTTGTGATGTAACTGG	ATTGGTGGAAAGTAACATCATCG
	EXPRESSED	GCTAAGAACGCTGGACCTAATG	TGGGTGTGCCTTTCTGAATG
SlVTE5-RNAi lines	RNAi-VTE5	CACCATGCAAGCTTTGGTGTGTG	CTAATAAGCAAAAGCCAATGATGCTAC
	35S-right	CCCACTATCCTTCGCAAG	
TILLING SlFOLK screening	external	TCATCTTTTAGAAGTGATATCCTAACC	ACACAATCCTGTGTTAGACTAACACAT
	internal	GAACAGAAAGCTTGTTCATATTAGCAT	ACATTGCCCATTTTCAGTAAGCATTTT

[a] 1-deoxy-D-xylulose-5-P synthase (DXS); geranylgeranyl pyrophosphate synthase (GGPS); geranylgeranyl diphosphate reductase (GGDR); 4-hydroxyphenylpyruvate dioxygenase (HPPD); homogentisate phytyl transferase (VTE2); 2,3-dimethyl-5-phytylquinol methyltransferase (VTE3); tocopherol cyclase (VTE1); γ-tocopherol-C-methyl transferase (VTE4); phytol kinase (VTE5); farnesol kinase (FOLK); chlorophyll synthase (CHLG); staygreen 1 (SGR1); pheophytinase (PPH); pheophorbide a oxygenase (PAO); homogentisate solanesyl transferase (HST); solanesyl-diphosphate synthase (SPS); phytoene synthase (PSY); phytoene desaturase (PDS); chloroplast-specific β-lycopene cyclase (LCYβ); chromoplast-specific β-lycopene cyclase (CYCβ); pale yellow petal/phytyl ester synthase (PYP/PES); clathrin adaptor complex medium subunit (CAC).

Supplementary Table S2. Tocopherol content and composition of *SlVTE5*-RNAi transgenic lines and *folk-1* mutant.

	α-tocopherol	β-/γ-tocopherol	δ-tocopherol	Total tocopherol
leaf				
WT	176.1 ± 51.4	24.9 ± 2.8	nd	201 ± 52.6
SlVTE5-RNAi#1	**28.7 ± 12.5**	**2.0 ± 1.9**	nd	**30.7 ± 12.1**
SlVTE5-RNAi#7	**21.2 ± 9.1**	**4.2 ± 1.7**	nd	**25.5 ± 10.7**
SlVTE5-RNAi#11	**22.1 ± 9.5**	**4.5 ± 1.5**	nd	**26.6 ± 10.9**
control	587.5 ± 60.0	6.8 ± 0.7	0.1 ± 0.1	594.4 ± 60.8
folk-1	611.7 ± 78.0	6.8 ± 1.0	0.2 ± 0.1	618.7 ± 79.1
Mature green fruit				
WT	53.6 ± 7.3	18.2 ± 4.1	nd	71.8 ± 10.1
SlVTE5-RNAi#1	**2.2 ± 3.4**	**11.0 ± 6.0**	nd	**13.1 ± 5.7**
SlVTE5-RNAi#7	**20.5 ± 6.2**	**2.5 ± 2.4**	nd	**23.0 ± 4.4**
SlVTE5-RNAi#11	**6.5 ± 7.3**	**3.7 ± 0.6**	nd	**10.3 ± 7.8**
control	103.8 ± 7.7	2.2 ± 0.6	0.1 ± 0.1	106.1 ± 8.3
folk-1	98.4 ± 11.9	2.8 ± 0.9	0.1 ± 0.1	101.3 ± 12.9
Ripe fruit				
WT	44.6 ± 25.3	43.8 ± 17.8	2.4 ± 2	90.8 ± 34.7
SlVTE5-RNAi#1	**3.2 ± 1.9**	**0.7 ± 1.5**	nd	**3.9 ± 3.2**
SlVTE5-RNAi#7	**3.3 ± 1.1**	**1.9 ± 3.3**	**0.2 ± 0.4**	**5.3 ± 4.7**
SlVTE5-RNAi#11	**6.3 ± 5.4**	**3.0 ± 5.1**	**0.3 ± 0.6**	**9.6 ± 7.6**
control	147.5 ± 11.1	15.9 ± 7.6	0.9 ± 0.4	164.2 ± 19.1
folk-1	115.8 ± 11.3	9.7 ± 4.1	0.4 ± 0.2	125.9 ± 15.6

Tocopherol content is showed as nmol. g^{-1} dry weight. The corresponding M4 segregating individuals homozygous for FOLK WT allele were used as control. Statistically significant differences between the wild-type (WT) and transgenic lines are indicated in bold terms (ANOVA/Dunnett's test, $P < 0.05$). nd, not detected.

Supplementary Table S3. Moles of prenyllipids found in *SlVTE5*-RNAi transgenic lines.

		Prenyllipid (nmol g^{-1} DW)					
		PC-8	PQ-9	PQH_2-9	tocopherol	FAPE	free phytol
Leaf	WT	57	653	322	200	290	90
	SlVTE5-RNAi [a]	**27**	508	339	**25**	**3000**	**400**
Mature green	WT	87	33	20	71	350	nm
	SlVTE5-RNAi [a]	**160**	**72**	21	**15**	450	nm
Ripe	WT	75	56	15	90	260	193
	SlVTE5-RNAi [a]	**150**	**120**	26	**6**	460	**1095**

[a] Mean value obtained from three transgenic lines. Significant differences between wild-type (WT) and transgenic lines are indicated in bold (ANOVA/Dunnett's test, $P < 0.05$). Plastochromanol-8 (PC-8), oxidized plastoquinone-9 (PQ-9), plastoquinol-9 (PQH_2-9), fatty acid phytyl ester (FAPE). nm, not measured.

Supplementary Table S4. Transcriptional profile of genes encoding isoprenoid metabolism-related enzymes.

	Leaf				Mature green fruits				Ripe fruits			
	WT	*SlVTE5*-RNAi#1	*SlVTE5*-RNAi#7	*SlVTE5*-RNAi#11	WT	*SlVTE5*-RNAi#1	*SlVTE5*-RNAi#7	*SlVTE5*-RNAi#11	WT	*SlVTE5*-RNAi#1	*SlVTE5*-RNAi#7	*SlVTE5*-RNAi#11
DXS(1)	1.00 ± 0.06	1.14 ± 0.15	1.12 ± 0.05	**1.53 ± 0.15**	1.00 ± 0.09	0.87 ± 0.09	0.87 ± 0.09	**0.58 ± 0.06**	1.00 ± 0.13	0.94 ± 0.18	**0.70 ± 0.07**	0.68 ± 0.14
GGPS(2)	1.00 ± 0.14	0.75 ± 0.08	1.06 ± 0.07	**1.80 ± 0.34**	1.00 ± 0.08	**1.43 ± 0.07**	1.23 ± 0.24	1.07 ± 0.07	1.00 ± 0.09	0.99 ± 0.06	**1.67 ± 0.22**	1.10 ± 0.17
GGDR	1.00 ± 0.07	0.84 ± 0.07	0.91 ± 0.04	**1.48 ± 0.20**	1.00 ± 0.17	0.96 ± 0.02	1.09 ± 0.14	**0.66 ± 0.06**	1.00 ± 0.27	1.21 ± 0.18	1.16 ± 0.16	0.68 ± 0.22
SPS	1.00 ± 0.12	**0.59 ± 0.06**	0.90 ± 0.04	0.92 ± 0.09	1.00 ± 0.10	**0.61 ± 0.02**	0.86 ± 0.13	**0.53 ± 0.05**	1.00 ± 0.11	1.20 ± 0.15	**1.36 ± 0.11**	1.17 ± 0.24
HST	1.00 ± 0.01	1.14 ± 0.12	1.03 ± 0.06	**1.37 ± 0.08**	1.00 ± 0.09	0.91 ± 0.09	1.20 ± 0.09	**0.75 ± 0.08**	1.00 ± 0.22	0.93 ± 0.12	**2.10 ± 0.26**	0.83 ± 0.13
HPPD(1)	1.00 ± 0.08	**0.70 ± 0.08**	**0.79 ± 0.01**	1.08 ± 0.05	1.00 ± 0.03	**1.28 ± 0.03**	**1.36 ± 0.13**	**1.09 ± 0.01**	1.00 ± 0.10	**1.38 ± 0.17**	**2.10 ± 0.37**	1.03 ± 0.17
HPPD(2)	1.00 ± 0.14	**0.52 ± 0.08**	0.80 ± 0.05	1.16 ± 0.05	1.00 ± 0.06	**1.74 ± 0.28**	**1.73 ± 0.18**	**1.25 ± 0.04**	1.00 ± 0.11	**1.55 ± 0.19**	**1.75 ± 0.37**	1.22 ± 0.21
VTE1	1.00 ± 0.06	1.06 ± 0.07	1.06 ± 0.03	**1.22 ± 0.02**	1.00 ± 0.10	0.98 ± 0.09	1.00 ± 0.03	**0.77 ± 0.05**	1.00 ± 0.19	1.11 ± 0.10	**1.57 ± 0.11**	0.98 ± 0.07
VTE2	1.00 ± 0.02	1.08 ± 0.06	**1.14 ± 0.07**	**1.28 ± 0.04**	1.00 ± 0.02	0.91 ± 0.10	**1.05 ± 0.02**	**0.83 ± 0.02**	1.00 ± 0.20	0.97 ± 0.10	1.26 ± 0.12	0.86 ± 0.08
VTE3(1)	1.00 ± 0.02	0.91 ± 0.07	**0.85 ± 0.02**	**0.96 ± 0.00**	1.00 ± 0.07	0.99 ± 0.04	1.08 ± 0.08	0.90 ± 0.02	1.00 ± 0.11	**1.23 ± 0.08**	**1.58 ± 0.16**	**1.39 ± 0.13**
VTE3(2)	1.00 ± 0.03	**0.73 ± 0.04**	**0.80 ± 0.03**	1.00 ± 0.02	1.00 ± 0.04	**0.84 ± 0.04**	1.01 ± 0.05	**0.71 ± 0.03**	1.00 ± 0.15	1.09 ± 0.03	**1.70 ± 0.27**	1.13 ± 0.10
VTE4	1.00 ± 0.09	0.86 ± 0.08	**0.71 ± 0.03**	**0.83 ± 0.02**	1.00 ± 0.10	0.86 ± 0.06	1.04 ± 0.09	**0.70 ± 0.02**	1.00 ± 0.10	0.89 ± 0.06	1.01 ± 0.10	1.10 ± 0.09
VTE5	1.00 ± 0.07	**0.19 ± 0.00**	**0.19 ± 0.01**	**0.14 ± 0.01**	1.00 ± 0.07	**0.25 ± 0.03**	**0.23 ± 0.02**	**0.19 ± 0.03**	1.00 ± 0.04	**0.06 ± 0.02**	**0.08 ± 0.01**	**0.06 ± 0.00**
FOLK	1.00 ± 0.09	0.92 ± 0.10	0.91 ± 0.04	1.07 ± 0.04	1.00 ± 0.07	0.86 ± 0.07	1.00 ± 0.07	**0.72 ± 0.04**	1.00 ± 0.14	**1.34 ± 0.11**	**1.93 ± 0.31**	1.28 ± 0.12
SGR1	1.00 ± 0.42	**0.56 ± 0.09**	**0.73 ± 0.29**	**0.54 ± 0.14**	1.00 ± 0.29	**1.83 ± 0.25**	**1.98 ± 0.16**	1.14 ± 0.10	1.00 ± 0.05	**1.55 ± 0.08**	**1.54 ± 0.25**	1.27 ± 0.15
CHLG	1.00 ± 0.01	**0.86 ± 0.04**	0.90 ± 0.07	**1.11 ± 0.04**	1.00 ± 0.02	0.97 ± 0.09	**1.17 ± 0.07**	**0.75 ± 0.05**	1.00 ± 0.12	1.16 ± 0.12	**1.88 ± 0.22**	1.04 ± 0.13
PAO	1.00 ± 0.04	0.77 ± 0.17	**0.90 ± 0.02**	**0.75 ± 0.07**	1.00 ± 0.12	**1.37 ± 0.09**	1.23 ± 0.11	0.97 ± 0.03	1.00 ± 0.01	1.04 ± 0.10	1.13 ± 0.07	1.04 ± 0.03
PPH	1.00 ± 0.06	0.79 ± 0.16	0.90 ± 0.02	0.84 ± 0.09	1.00 ± 0.05	1.07 ± 0.11	0.81 ± 0.09	**0.71 ± 0.03**	1.00 ± 0.17	**1.39 ± 0.07**	**1.78 ± 0.43**	1.20 ± 0.28
PYP/PES(1)	1.00 ± 0.10	**1.48 ± 0.21**	**1.56 ± 0.05**	**1.92 ± 0.05**	1.00 ± 0.14	0.96 ± 0.08	1.06 ± 0.15	**0.66 ± 0.05**	1.00 ± 0.11	1.50 ± 0.33	1.11 ± 0.01	0.92 ± 0.17
PES(2)	1.00 ± 0.11	1.11 ± 0.14	1.16 ± 0.10	1.09 ± 0.06	1.00 ± 0.01	1.14 ± 0.08	**1.25 ± 0.09**	1.02 ± 0.05	1.00 ± 0.12	1.02 ± 0.10	1.20 ± 0.08	1.09 ± 0.13
PSY(1)	1.00 ± 0.10	**0.37 ± 0.04**	**0.58 ± 0.07**	1.19 ± 0.12	1.00 ± 0.12	**1.89 ± 0.31**	**2.13 ± 0.37**	**1.30 ± 0.07**	1.00 ± 0.06	**1.41 ± 0.15**	**0.79 ± 0.08**	1.12 ± 0.13
PSY(2)	1.00 ± 0.03	**0.44 ± 0.05**	**0.58 ± 0.02**	1.10 ± 0.13	1.00 ± 0.04	**0.84 ± 0.05**	0.92 ± 0.07	0.87 ± 0.15	1.00 ± 0.07	**1.45 ± 0.21**	1.07 ± 0.08	1.07 ± 0.27
PDS	1.00 ± 0.08	**1.22 ± 0.05**	**1.29 ± 0.10**	**1.37 ± 0.02**	1.00 ± 0.06	**0.79 ± 0.04**	0.90 ± 0.04	**0.72 ± 0.05**	1.00 ± 0.05	1.01 ± 0.15	0.84 ± 0.22	1.09 ± 0.08
LCY-β	1.00 ± 0.06	**0.65 ± 0.03**	**0.70 ± 0.04**	1.00 ± 0.07	1.00 ± 0.05	1.00 ± 0.03	1.12 ± 0.12	**0.86 ± 0.06**	1.00 ± 0.05	1.16 ± 0.11	**0.85 ± 0.06**	0.81 ± 0.11
CYC-β	1.00 ± 0.09	**2.22 ± 0.27**	**1.57 ± 0.08**	**2.61 ± 0.29**	1.00 ± 0.08	**0.54 ± 0.08**	**0.70 ± 0.13**	**0.79 ± 0.06**	1.00 ± 0.05	**1.34 ± 0.14**	**1.54 ± 0.16**	**1.61 ± 0.16**

Values represent means from at least three biological replicates. The expression data shown represent fold-change compared to control wild-type (WT) in the respective organ. Significant differences were determined according to a permutation test ($P<0.05$) and indicate in bold. 1-deoxy-D-xylulose-5-P synthase (DXS); geranylgeranyl pyrophosphate synthase (GGPS); geranylgeranyl diphosphate reductase (GGDR); 4-hydroxyphenylpyruvate dioxygenase (HPPD); tocopherol cyclase (VTE1); homogentisate phytyl transferase (VTE2); 2,3-dimethyl-5-phytylquinol methyltransferase (VTE3); γ-tocopherol-C-methyl transferase (VTE4); phytol kinase (VTE5); farnesol kinase (FOLK); staygreen 1 (SGR1); chlorophyll synthase (CHLG); pheophytinase (PPH); pheophorbide a oxygenase (PAO); pale yellow petal/phytyl ester synthase (PYP/PES); homogentisate solanesyl transferase (HST); solanesyl-diphosphate synthase (SPS); phytoene synthase (PSY); phytoene desaturase (PDS); chloroplast-specific β-lycopene cyclase (LCYβ); chromoplast-specific β-lycopene cyclase (CYCβ).

Supplementary Table S5. Changes in fatty acid-derived lipids in leaves of *SlVTE5*-RNAi transgenic lines compared with wild-type.

		SlVTE5-RNAi		
Relative amounts	WT	# 1	#7	#11
MGDG-18:3/16:3	1.00 ± 0.06	0.92 ± 0.07	0.93 ± 0.03	0.96 ± 0.03
MGDG-18:3/18:3	1.00 ± 0.05	0.93 ± 0.08	**0.91 ± 0.02**	0.97 ± 0.03
DGDG-18:3/18:3	1.00 ± 0.05	**0.90 ± 0.07**	**0.89 ± 0.03**	**0.91 ± 0.04**
DGDG-18:3/16:0	1.00 ± 0.04	**0.90 ± 0.09**	**0.89 ± 0.06**	0.96 ± 0.03
DGDG-18:2/16:0	1.00 ± 0.08	1.09 ± 0.13	1.00 ± 0.07	0.92 ± 0.03
MGDG-18:2/18:3	1.00 ± 0.05	0.99 ± 0.13	0.91 ± 0.09	0.88 ± 0.01
DGDG-18:3/16:3	1.00 ± 0.07	**0.84 ± 0.09**	**0.88 ± 0.06**	**0.88 ± 0.05**
MGDG-18:3/16:1	1.00 ± 0.10	0.98 ± 0.14	0.95 ± 0.10	0.99 ± 0.09
DGDG-18:1/18:3	1.00 ± 0.02	0.93 ± 0.06	0.92 ± 0.05	**0.85 ± 0.06**
DGDG-18:3/20:3	1.00 ± 0.15	0.78 ± 0.21	0.81 ± 0.11	**0.76 ± 0.04**
DGDG-18:0/18:3	1.00 ± 0.11	0.85 ± 0.12	0.85 ± 0.16	**0.75 ± 0.06**
PE-18:2/16:0	1.00 ± 0.08	1.08 ± 0.04	1.07 ± 0.08	1.10 ± 0.10
PE-18:3/16:0	1.00 ± 0.06	0.92 ± 0.13	0.92 ± 0.20	0.92 ± 0.11
PE-18:2/18:2	1.00 ± 0.27	1.19 ± 0.17	1.11 ± 0.18	**1.38 ± 0.20**
DAG-18:0/18:0	1.00 ± 0.17	0.93 ± 0.25	0.79 ± 0.10	0.82 ± 0.22
DAG-18:0/16:0	1.00 ± 0.16	0.93 ± 0.26	0.77 ± 0.09	0.83 ± 0.23
DAG	1.00 ± 0.17	0.93 ± 0.25	0.79 ± 0.09	0.82 ± 0.22

Data were normalized to sample dry weight and expressed relative to wild type (WT) in each tissue. Values are represented as means ± SD. Terms in bold indicate a statistically significant difference by ANOVA/Dunnett's test ($P < 0.05$). Monogalactosyldiacylglycerol (MGDG); digalactosyldiacylglycerol (DGDG); phosphotidylethanolamine (PE); diacylglycerol (DAG).

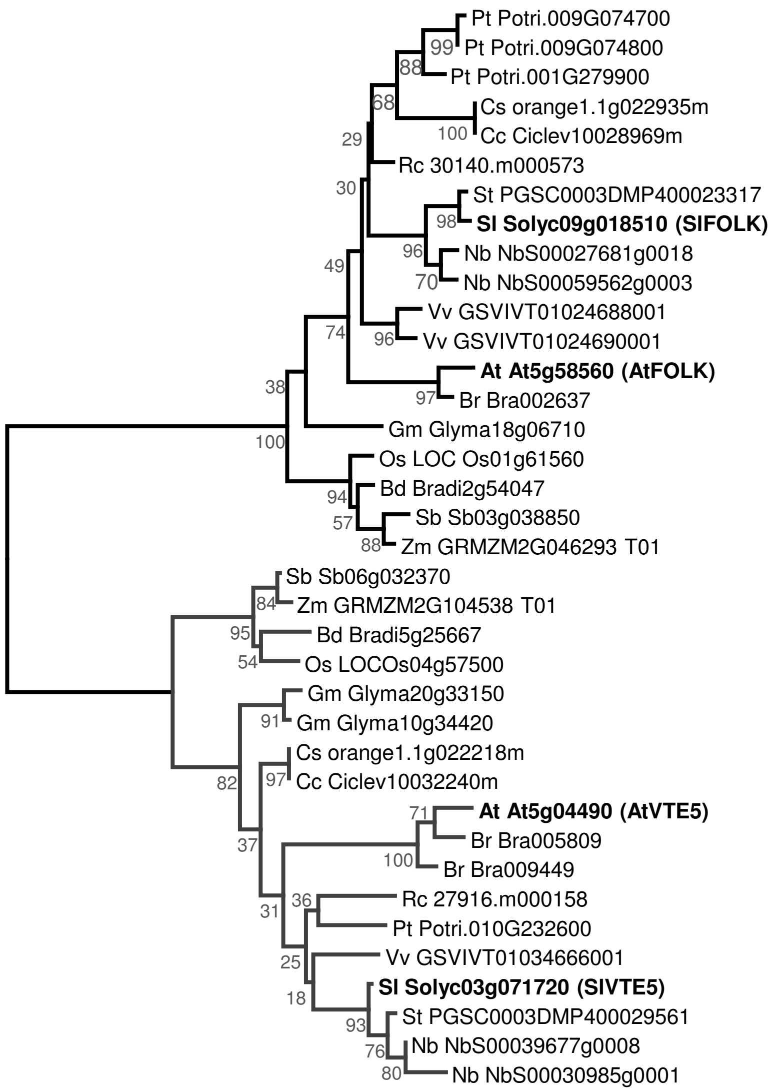

Supplementary Fig. S1 Phylogenetic analysis VTE5 and FOLK proteins. Neighbor-joining phylogeny of VTE5 and FOLK homologs in flowering plants, which were identified by Blastp searches using AtVTE5 as the query against the Phytozome database (http://www.phytozome.net). Br, *Brassica rapa;* Bd, *Brachypodium distachyon*; Cc, *Citrus clementina*; Cs, *Citrus sinensis*; Pt, *Populus trichocarpa;* Nb, *Nicotiana benthamiana*; St, *Solanum tuberosum;* Sb, *Sorghum bicolor;* Zm, *Zea mays;* Os, *Oryza sativa;* Vv, *Vitis vinifera;* Rc, *Ricinus communis.* Gene numbers following names are based on those listed at Phytozome, with the exception of Arabidopsis ID.

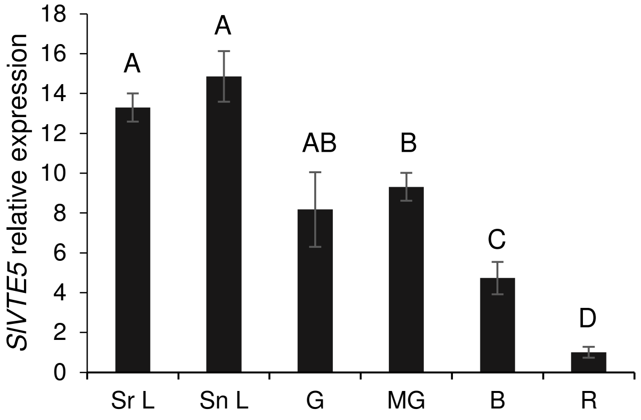

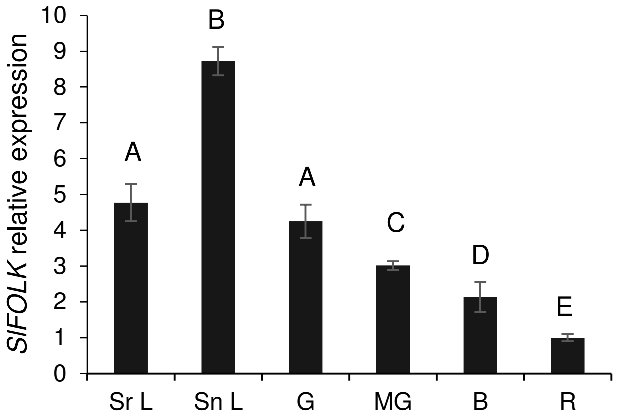

Supplementary Fig. S2 Expression of *SlVTE5* and *SlFOLK*. Relative expression were measured by qPCR in source (SrL) and sink (SnL) leaves, green (G), mature green (MG), breaker (B) and ripe (R) fruits in at least three biological replicates. The means were calculated from two technical replicates and normalized against R fruit samples. Statistically significant differences (permutation test, $P < 0.05$) are indicated with different letters.

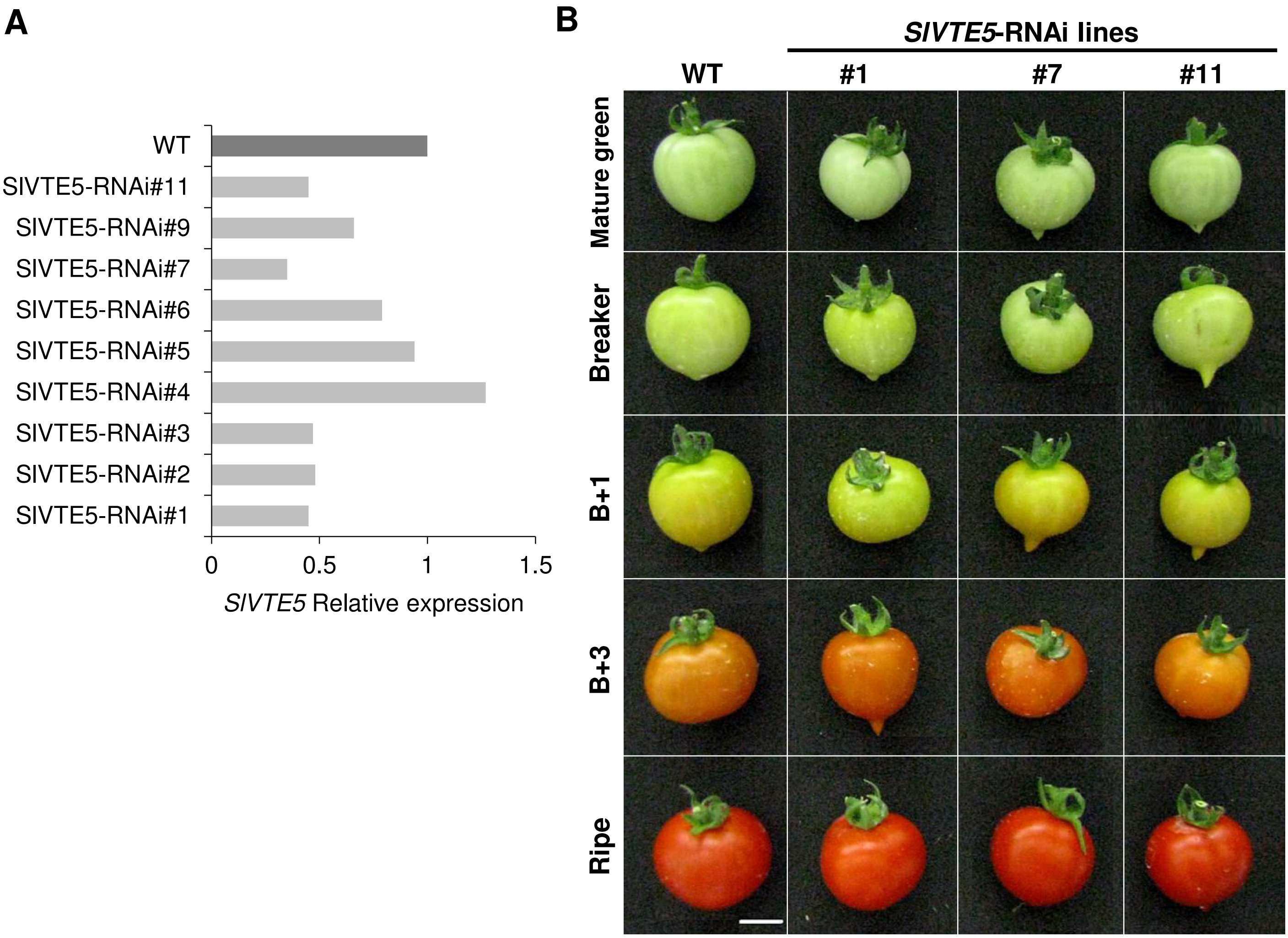

Supplementary Fig. S3 Screening of primary transformants and fruit phenotype of *SlVTE5*-RNAi transgenic lines. (A) *SlVTE5* mRNA levels in source leaves from T0 transformants. Bars represent the mean of three technical replicates. Values were expressed as relative abundance of mRNA compared to wild-type (WT). (B) Representative T1 fruits of *SlVTE5*-RNAi transgenic lines at mature green, breaker, one day after breaker (B+1), three days after breaker (B+3) and ripe (B+6) stages. Scale bar = 1 cm.

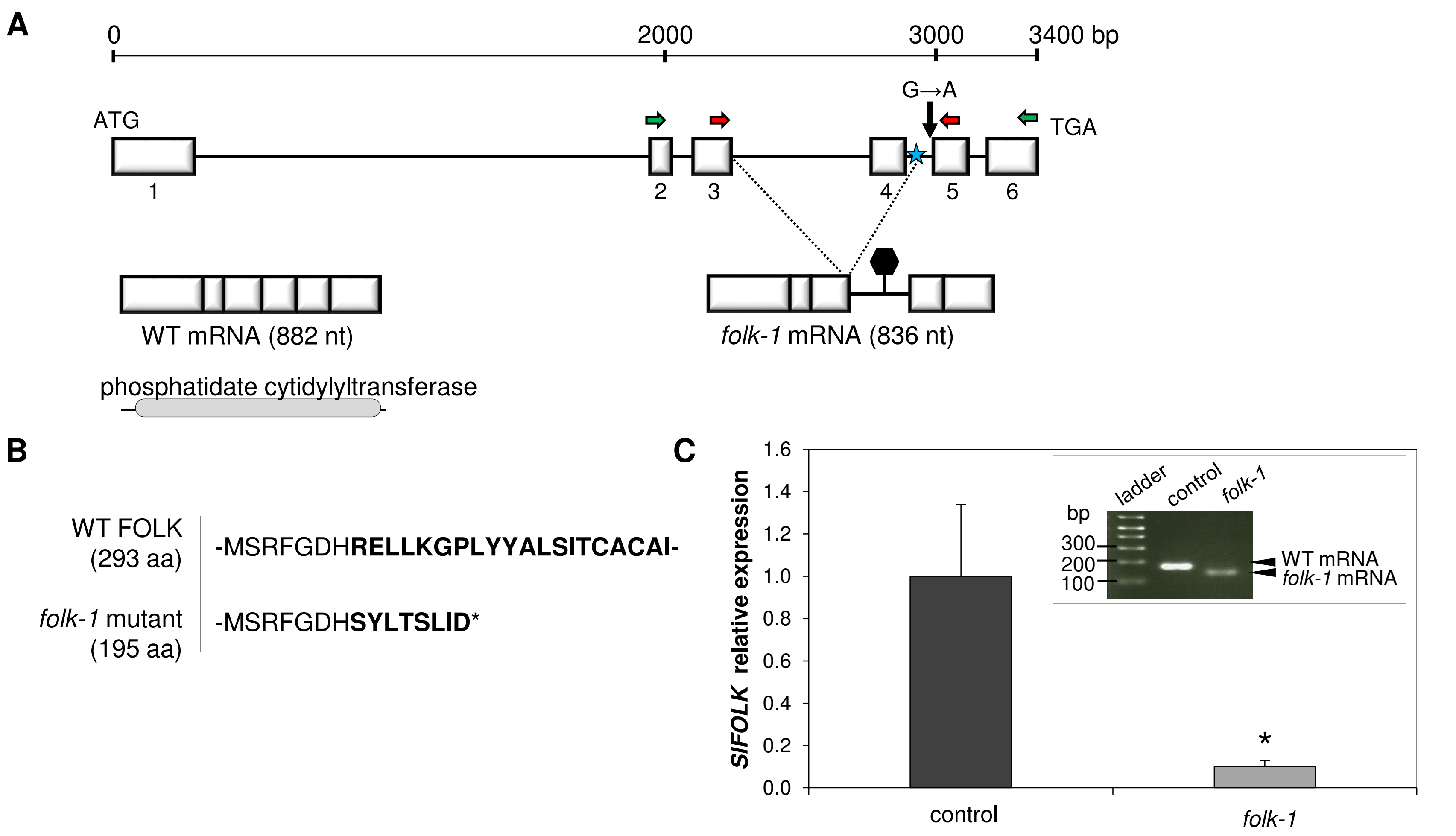

Supplementary Fig. S4 Analysis of *folk-1* mutation**.** (A) Schematic diagram of *SlFOLK* (Solyc09g018510) splicing pattern showing the fully spliced wild-type (WT) and *folk-1* mutant mRNA. The open boxes and solid lines represent exons and introns, respectively. The green arrows indicate the position of the primers used for TILLING screening and the red arrows indicate intron-flanking primers used for qPCR assay. The position of the G to A substitution (G2976A) is indicated in the 3' consensus splicing site of intron 4. The star denotes the 3' cryptic splicing site. The *folk-1* mutant mRNA generates a premature stop codon indicated by a black hexagon, which presumably affects the phosphatidate cytidylyltransferase domain. (B) Partial amino acid sequences deduced from WT and abnormal cryptic site transcripts. Frameshift in predicted amino acid sequence is indicated in bold. (C) qPCR analysis of *SlFOLK* expression in leaves of M4 plants homozygous for *folk-1* allele. The corresponding segregating individuals homozygous for *FOLK* wild-type allele were used as control. Data are means ± SEM of three biological replicates. Asterisk denotes statistically significant differences (permutation test, $P < 0.05$). The insets shows agarose gel electrophoresis of *SlFOLK* PCR amplicons obtained from control and *folk-1* plants. Nt, nucleotide; bp, base pair; aa, amino acid.

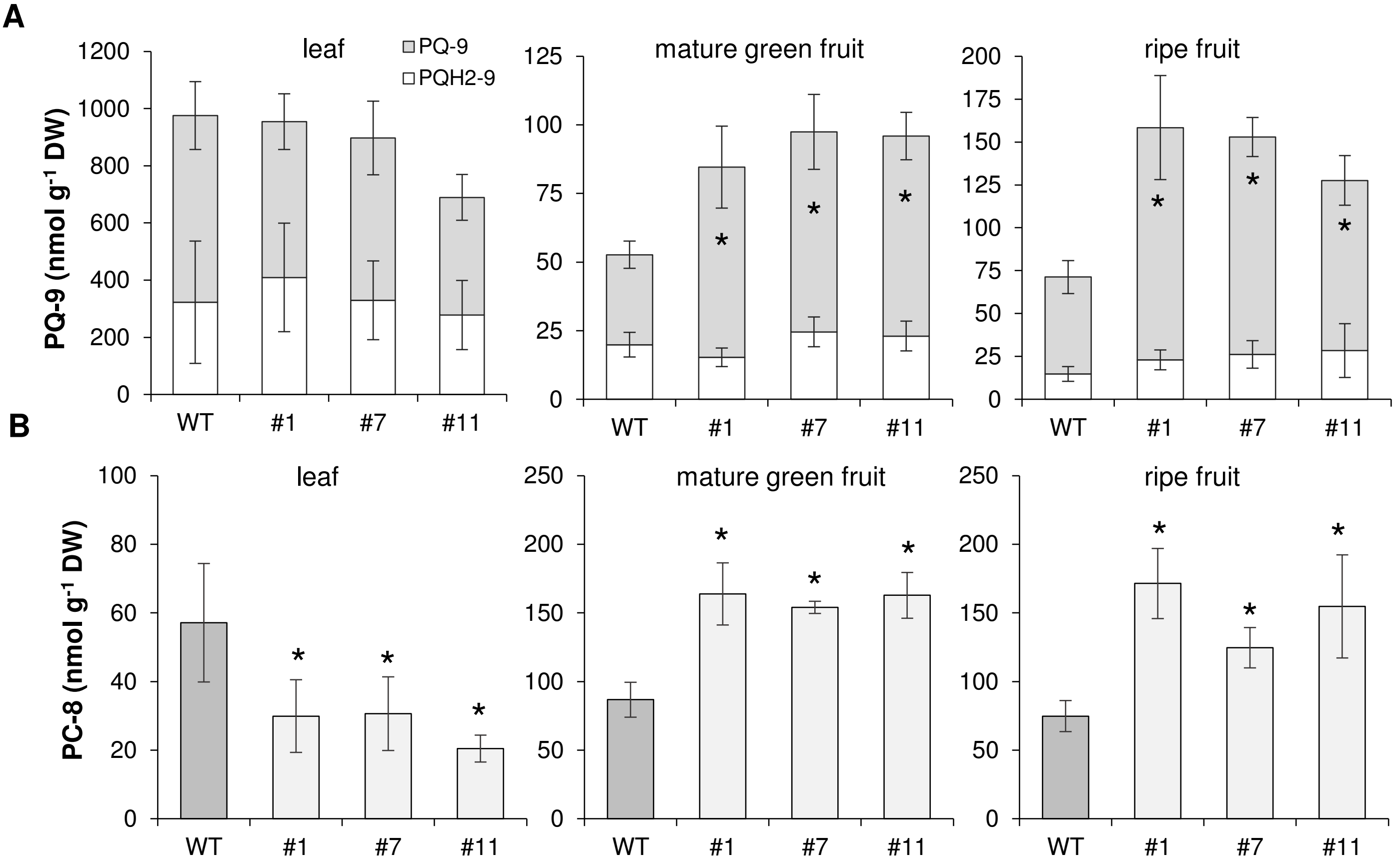

Supplementary Fig. S5 Plastoquinone (PQ-9) and plastochromanol (PC-8) levels in *SlVTE5*-RNAi transgenic lines. Prenylquinone were measured in leaves, mature green and ripe fruits of *SlVTE5*-RNAi lines. (A) Total PQ-9 content; bars indicate the fraction of oxidized (PQ-9) and reduced (PQH_2-9) forms. (B) PC-8 content. Data represent the mean ± SD of five biological replicates. Asterisk denotes significant differences between the wild-type (WT) and the transgenic lines (ANOVA/Dunnett's test, $P < 0.05$). DW, dry weight.

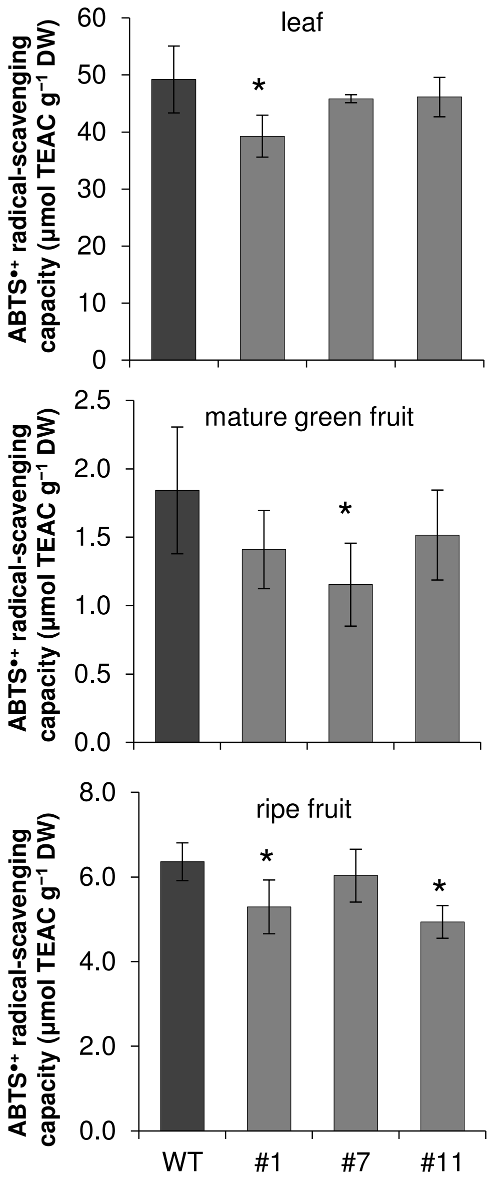

Supplementary Fig. S6 Trolox equivalent antioxidant capacity (TEAC) in leaves and fruits of *SlVTE5*-RNAi transgenic lines. Data represent the mean ± SD of five biological replicates. Measurements are from three technical replicates. Asterisk denotes significant differences between the wild-type (WT) and the transgenic lines (ANOVA/Dunnett's test, $P < 0.05$). DW, dry weight.

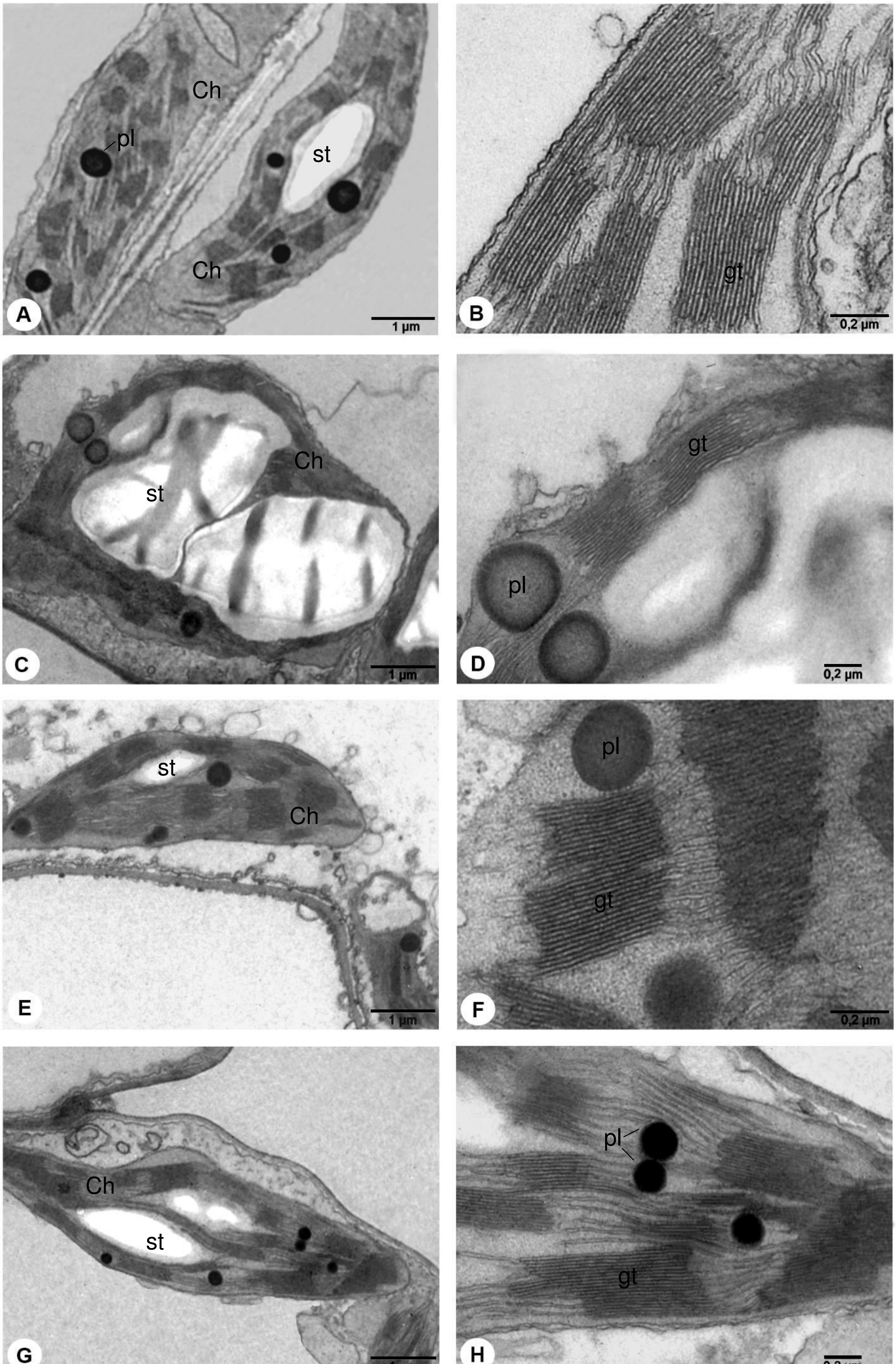

Supplementary Fig. S7 Chloroplast ultrastructure resulting from *SlVTE5* downregulation. Transmission electron micrographs of the first leaflet from the first fully expanded leaf at the middle of day-light (16-week-old plant). (A,B) wild-type, (C,D) *SlVTE5*-RNAi#1, (E,F) *SlVTE5*-RNAi#7 and (G,H) *SlVTE5*-RNAi#11. Ch: chloroplast; st: starch granule; pl: plastoglobule; gt: grana thylakoids.
